# The Possibility of Using *Paulownia elongata* S. Y. Hu × *Paulownia fortunei* Hybrid for Phytoextraction of Toxic Elements from Post-Industrial Wastes with Biochar

**DOI:** 10.3390/plants10102049

**Published:** 2021-09-29

**Authors:** Kinga Drzewiecka, Monika Gąsecka, Zuzanna Magdziak, Sylwia Budzyńska, Małgorzata Szostek, Przemysław Niedzielski, Anna Budka, Edward Roszyk, Beata Doczekalska, Marta Górska, Mirosław Mleczek

**Affiliations:** 1 Department of Chemistry, Faculty of Forestry and Wood Technology, Poznań University of Life Sciences, Wojska Polskiego 75, 60-625 Poznań, Poland; monika.gasecka@up.poznan.pl (M.G.); zuzanna.magdziak@up.poznan.pl (Z.M.); sylwia.budzynska@up.poznan.pl (S.B.); 2 Department of Soil Science, Environmental Chemistry and Hydrology, University of Rzeszów, Zelwerowicza 8b, 35-601 Rzeszów, Poland; mszostek@ur.edu.pl; 3 Department of Analytical Chemistry, Faculty of Chemistry, Adam Mickiewicz University in Poznań, Uniwersytetu Poznańskiego 8, 61-614 Poznań, Poland; pnied@amu.edu.pl; 4 Department of Mathematical and Statistical Methods, Poznań University of Life Sciences, Wojska Polskiego 28, 60-637 Poznań, Poland; anna.budka@up.poznan.pl; 5 Department of Wood Science and Thermal Techniques, Faculty of Forestry and Wood Technology, Poznań University of Life Sciences, Wojska Polskiego 38/42, 60-637 Poznań, Poland; edward.roszyk@up.poznan.pl (E.R.); marta.gorska@up.poznan.pl (M.G.); 6 Department of Chemical Wood Technology, Faculty of Forestry and Wood Technology, Poznań University of Life Sciences, Wojska Polskiego 38/42, 60-637 Poznań, Poland; beata.doczekalska@up.poznan.pl

**Keywords:** hybrid, Oxytree, phytoextraction, trace elements, uptake

## Abstract

The potential of the *Paulownia* hybrid for the uptake and transport of 67 elements along with the physiological response of plants cultivated in highly contaminated post-industrial wastes (flotation tailings—FT, and mining sludge—MS) was investigated. Biochar (BR) was added to substrates to limit metal mobility and facilitate plant survival. *Paulownia* could effectively uptake and translocate B, Ca, K, P, Rb, Re and Ta. Despite severe growth retardation, chlorophyll biosynthesis was not depleted, while an increased carotenoid content was noted for plants cultivated in waste materials. In *Paulownia* leaves and roots hydroxybenzoic acids (C6-C1) were dominant phenolics, and hydroxycinnamic acids/phenylpropanoids (C6-C3) and flavonoids (C6-C3-C6) were also detected. Plant cultivation in wastes resulted in quantitative changes in the phenolic fraction, and a significant drop or total inhibition of particular phenolics. Cultivation in waste materials resulted in increased biosynthesis of malic and succinic acids in the roots of FT-cultivated plants, and malic and acetic acids in the case of MS/BR substrate. The obtained results indicate that the addition of biochar can support the adaptation of *Paulownia* seedlings growing on MS, however, in order to limit unfavorable changes in the plant, an optimal addition of waste is necessary.

## 1. Introduction

The genus *Paulownia* includes mainly herbs with only a few woody plants. Fast-growing, deciduous trees of this genus naturally occur in Southeast Asia, but some species have been successfully introduced in Poland and can be found in the warmest areas in the country, i.e., *Paulownia tomentosa* (Thunb.) Steud. (known as the princess tree) and *Paulownia fortunei* (Seem.) Hemsl. (commonly called the dragon tree). Both of them grow up to 20 m tall, and their crowns are typically spherical-shaped. The *Paulownia* tree is difficult to propagate since its seeds are highly vulnerable to soil fungi. Therefore, in vitro reproduction techniques are implemented to produce seedlings. To maximize the advantages of various species, some new artificial species and their varieties have been developed, e.g., *Paulownia* “Shan Tong” and *Paulownia* “Cotta Vista 2” (*Paulownia tomentosa* and *Paulownia fortunei* hybrid) [1], *Paulownia* Clon in Vitro 112 (*Paulownia elongata* × *fortunei* hybrid) developed by the Spanish In Vitro S.L. company. Trees of the latest clone assimilate more CO_2_ than other taxa of the *Paulownia* genus, and consequently produce more O_2_, earning them the name of “Oxytree”, a commercial name given by Carbon Solutions Global Ltd. in 2016 [2]. The extremely fast-growing Oxytree was introduced in Poland in 2015, and is considered as an alternative in biomass, wood, pulp production, as well as the recovery processes of soils exploited by mining. Thus, *Paulownia* plantations have become increasingly popular [3].

Along with poplar and willow, plants of the *Paulownia* genus are usually described as good candidates for phytoremediative purposes due to their tolerance to high concentrations of metals, rapid growth, and above all, high biomass production [4]. This aspect has been described for selected hybrids and most often concerns assisted phytoremediation using particular agents added to substrate [5,6,7]. Trees of the genus *Paulownia* may be suitable for metal phytoextraction due to their belonging to the C4 plant group, whose highly reductive internal environment of cells prevents the oxidative conversion of metals [8]. As part of numerous studies in this area, significant differences in the phytoextraction of harmful elements have been identified. In soil heavily contaminated with copper compounds, the *Paulownia elongata* × *fortunei* hybrid showed good resistance and reduced the Cu content in the soil by 17% [3]. It was found that the addition of EDTA caused a significantly higher uptake of metals compared to citrate [9]. Attention has also been paid to the use of fungi or peat as growth-stimulating agents influencing the phytoextraction potential of *Paulownia* [10,11]. Further, *Paulownia* has so far only been investigated to a limited extent on mining waste materials and there is lack of extensive research on the accumulation of noble or rare earth elements. However, bioconcentration and translocation factors exceeding one for common metallic pollutants as Cd, Cu, Pb and Zn, make *Paulownia* an increasingly attractive research subject [10]. Wang et al. [12] showed that during revegetation, the immobility and bioavailability of heavy metals were enhanced in the rhizosphere soils of *Paulownia fortunei*.

An effective reduction of metal load in soil using *Paulownia tomentosa*, almost 2-fold higher than for *Cytisus scoparius* or *Populus alba* was documented [13]. The high potential of two lines of *Paulownia* was also described [14] with clear differentiation in phytoextraction of Pb and Zn. Nevertheless, despite valuable morphological features and significantly higher phytoremediative abilities than most woody non-hyperaccumulators, the practical use of *Paulownia* is clearly limited due to non-native trees are now prominently on the lists of invasive plants in many parts of the world [15]. Possibilities for the use of the *Paulownia* genus can be seen in hybrids such as *Paulownia elogata × fortunei* that produce non-viable seeds and limiting the invasion risks [16].

Plant growth on contaminated soil is already problematic at the adaptation stage when the plant experiences contact with high concentrations of toxic elements. A promising solution undertaken in several works is the use of biochar. Many studies have reported that biochar has been effectively used to immobilize metal(loid)s in contaminated soils and influences their bioavailability and bioaccessibility for plants [17,18]. Biochar amended bioremediation is one of the critical remedial technologies to remediate soils contaminated by metal(loid)s. Biochar-enhanced phytoremediation shows excellent potential to immobilize cationic metal(loid)s in mine wastes and tailing soils, particularly those with high acidity. The moderate specific surface area of biochar and a pH higher than 9.0 may help plants to survive in new unfavorable growth conditions by reducing the concentration of toxic elements in the rhizosphere and their gradual dosing. Biochar may also improve soil fertility and revegetation and create a suitable environment for soil microbial diversity. The potential benefits of biochar for phytoremediation are (1) physical adsorption of cationic metal(loid)s from soil pore water; (2) (co)precipitation with phosphate, carbonates, silicate, and chloride, e.g., the formation of pyromorphite; (3) complexation with functional groups on the surface of biochar; and (4) the release of nutrients such as N, P, K, Ca. Processes (1)–(3) can reduce the bioavailable metal concentrations in soil pore water and further minimize phytotoxicity. Process (4) can produce nutrients for plant roots and microorganisms in the rhizosphere, which is a crucial point in the development of cost-effective remediation strategies [19].

The search for plants capable of adaptation and growth on post-industrial waste is necessary due to the need for the decontamination of polluted matrices and their use in phytomining. The use of species recognized at present as invasive, although in the context of progressive climate change potentially non-invasive species in the future, seems rational, providing these plants are characterized by high resistance and accumulation of pollutants while growing on waste with moderate concentrations of toxic elements.

The aim of this study was to characterize the potential of the *Paulownia elongata* × *Paulownia fortunei* hybrid to uptake 67 elements (including rare earth elements (REEs) and noble elements (NEs) together with an estimation of the physiological response of plants cultivated in highly contaminated post-industrial waste materials. Due to the high concentrations of metals and metalloids in the applied waste materials, biochar was used to assess its use as a factor limiting metal mobility and availability for plants, and ultimately facilitating the survival of *Paulownia* seedlings.

## 2. Materials and Methods

### 2.1. Plant Material

Oxytree (*Paulownia elongata* S. Y. Hu × *Paulownia fortunei* (Seem.) Hemsl.) seedlings with the trade name of in Vitro 112, were obtained from an authorized supplier, Oxytree Solutions Poland Ltd. (formerly Carbon Solutions Poland Ltd.). Seedlings (6–8-month-old) were used after their preliminary selection from a population of 10,000 specimens according to similar biomass (14–16 g), height (15 cm) and leaf count (5 pcs).

### 2.2. Experiment Design

Five experimental systems were prepared in hydroponic pots (23 × 23 cm, diameter × height) as follows: (1) control—6.1 kg of reference soil, (2) FT—4.2 kg of flotation tailings, (3) FT/BR—3.99 kg of FT and 0.21 kg of biochar (BR), (4) MS—4.6 kg of mining sludge and (5) MS/BR—4.37 kg of MS and 0.23 kg of BR. Biochar addition was set at 5% (w/w) based on preliminary trials where its application increased the porosity of waste materials and eventually led to undisturbed water flow in the substrate. Biochar was obtained from miscanthus (*Miscanthus gigantheus*). The lignocellulosic material was ground with a roller mill and sieved. The crushed plants were subjected to pyrolysis and carbonization in a chamber reactor in an oxygen free atmosphere by heating up to 600 °C at the temperature rate of 3 °C min^−1^ and then holding for 1 h. The pore structure of the biochar was characterized by the nitrogen adsorption-desorption method at 77.4 K in a sorptometer ASAP Micromeritics 2020. Prior to gas adsorption measurements, the biochar was degassed at 300 °C in a vacuum condition for 24 h. The Brunauer-Emmett-Teller (BET) surface area was calculated from the isotherms using the BET equation. The pH of the aqueous biochar solution was also determined. The mixtures of waste materials and BR (characterized by the specific surface area of 50.1 m^2^ g^−1^) were carefully blended using a POLYMIX PX-SR 90 D stirrer (KINEMATICA AG, Littau-Luzern, Switzerland). Detailed characteristics of the resulting experimental substrates are shown in Tables 1 and 2 in the Results section.

Each experimental system combined 6 plants growing one plant per pot. The experiment was set up on 5th April 2018 and terminated on 27th September 2019. Plants were cultivated in a ventilated greenhouse of the Botanical Garden administered by the Adam Mickiewicz University in Poznań. The humidity of substrates was continuously measured, and the plants were constantly watered with tap water using an automatic irrigation system to maintain a constant humidity. The temperature was controlled by a mechanical ventilation system and automatic data loggers recording ambient parameters on an hourly basis. Mean temperature, air relative humidity and concentration of CO_2_ were 22.8 °C, 50.5% and 459 ppm, respectively.

After the growing season of 2018, 3 randomly selected plants from each experimental system were harvested, gently washed with ultrapure water (Milli-Q, Millipore, Saint Luis, MO, USA) and divided into roots, stems and leaves. The biomass of separated organs was determined by weighing. For physiological and biochemical investigations, fresh organs were in situ frozen in liquid nitrogen and stored at −80 °C till analyses. Before extraction, each plant sample was homogenized in liquid nitrogen. For the elemental investigations, collected tissue was dried at 55 ± 2 °C to a constant weight using an electric oven (SLW 53 STD, Pol-Eko, Wodzisław Śląski, Poland). The dry material was ground using an SM 200 Cutting Mill (Retsch GmbH, Haan, Germany) until a powder fraction was obtained. The remaining 3 plants from each group were left for the next growing season (2019), and the harvested plants were investigated for biomass parameters and metal accumulation.

### 2.3. Analysis of Experimental Substrates

Substrate samples were air dried and sieved through a 2 mm sieve. Particle size distribution was performed with the laser diffraction method using the Laser Particle Sizer ANALYSETTE 22 (Fritsch, Idar-Oberstein, Germany). pH was analyzed in a 1:2.5 substrate-water suspension using a Hanna Instruments (Nusfalaucity, Romania) 4221 pH-meter. Electrical conductivity (EC) was analyzed in a 1:5 substrate-water suspension with a HI 2316 EC-meter by Hanna Instruments (Nusfalau, Romania). Total carbon (TC), nitrogen (Nt) and sulfur (St) were determined by the dry combustion method using the auto analyzer Vario El CUBE (Elementar Analysensysteme GmbH, Langenselbold, German) [20]. Soil organic carbon (SOC) was determined using the Walkley-Black procedure [20]. Inorganic carbon (SIC) was calculated as a difference between total carbon (TC) and organic carbon (SOC). Humic substances (HS)—humic acids (C_HA_), fulvic acids (C_FA_) and humins (C_HUMIN_) were determined according to Kononowa [21]. The base cations (Ca^2+^, Mg^2+^, K^+^, Na^+^) were determined by an inductively coupled plasma optical emission spectrometer (ICP-OES 5110, Agilent, Santa Clara, CA, USA) following the extraction with 1 M ammonium acetate. Cation exchange capacity (CEC) and base cation saturation ratio were calculated according to Culman et al. [22].

### 2.4. Analysis of Elements in Soil and Plant Samples

In the experimental substrates and plant organs, 67 elements were analyzed as follows: Al, As, B, Ba, Be, Bi, Ca, Cd, Co, Cr, Cs, Cu, Fe, Ga, Ge, Hf, Hg, In, K, Li, Mg, Mn, Mo, Na, Ni, P, Pb, Rb, Re, Sb, Se, Si, Sn, Sr, Ta, Te, Th, Ti, Tl, V, W, Zn, Zr, rare earth elements (REEs): Ce, Dy, Er, Eu, Gd, Ho, La, Lu, Nd, Pr, Sc, Sm, Tb, Tm, Y and Yb and noble elements (NEs): Ag, Au, Ir, Pd, Rh, Ru, Os, Pt.

For the fractionation of elements in all experimental substrates, the four steps sequential extraction proposed by the Commission of the European Communities Bureau of Reference (BCR) procedure [23] was performed as follows:

Step 1—Exchangeable/extractable fraction (F1): 40 mL 0.11 M acetic acid was added to a 100 mL centrifuge tube containing 1 g of dry soil sample and sieved through a 2-mm grid. The samples were then shaken at room temperature for 16 h. The supernatant and solid were decanted and kept for further analysis. The residual solid was rinsed twice with distilled water (2 × 10 mL) by shaking for 15 min. After centrifugation, the liquid was decanted and discarded.
Step 2— Reducible fraction bound Fe–Mn oxides (F2): 40 mL 0.5 M hydroxyloammonium chloride solution was added to the centrifuge tube containing the residue from step 1. The samples were shaken once more at room temperature for 16 h. The samples were then centrifuged and treated as in step 1.
Step 3— Oxidizable fraction bound to organic matter (F3): 10 mL 8.8 M hydrogen peroxide solution was added to the Step 2 residue. The contents were digested first at room temperature for 1 h, then at 85 °C in a water bath until approximately 1 mL of solution was obtained. Then 50 mL 1 M ammonium acetate was added and shaken for 16 h at room temperature. The supernatant was collected after centrifugation and kept for further analysis. The solid was rinsed as before.
Step 4— Residual fraction (F4): The residue remaining at the end of step 3 was digested in aqua regia solution and the concentration of REEs was determined using an inductively coupled plasma optical emission spectrometer (ICP-OES 5110, Agilent, USA).

#### 2.4.1. Sample Processing

Samples of dry material (0.300 ± 0.001 g) were digested with concentrated nitric acid (65%; Sigma-Aldrich, Saint Louis, MO, USA) in Teflon containers using a closed microwave sample preparation system (Mars 6 Xpress, CEM, Matthews, NC, USA). After digestion, the samples were diluted with Milli-Q water (Merck Millipore, Darmstadt, Germany) to a final volume of 15 mL and filtered (Qualitative Filter Papers Whatman, Grade 595). Concentration of all determined elements is expressed as mg kg^−1^ DW.

#### 2.4.2. Instruments and Quality Control

The inductively coupled plasma optical emission spectrometry (Agilent 5110 ICP-OES, Agilent, USA) was used for analysis. The following conditions of analytical procedure were set: radio frequency (RF) power 1.2 kW; 0.7, 1.0 and 12.0 L min^−1^, respectively for nebulizer gas, auxiliary gas and plasma gas flows; detector charge coupled device (CCD) temperature −40 °C; the time of signal accusation 5 s for 3 replicates. The detection limit for all elements determined was set (as 3-sigma criteria) at the level of 0.01–0.09 mg kg^−1^ of dry weight (DW). The uncertainty for the total analytical procedure (including sample preparation, for uncertainty budget calculation, the coverage factor k = 2 was used) was at the level of 20%. The recovery for certified reference materials analysis (CRM NCSDC 73349—bush branches and leaves, CRM S-1 – loess soil, CRM 2709 – soil) was acceptable (80–120%) for most of the determined elements.

### 2.5. Determination of Arsenic Species in Soil and Plant Samples

#### 2.5.1. Preparation of Samples

Dry samples (0.300 ± 0.001 g) were extracted in a glass flask with 10 mL of phosphoric acid (1 M) in an ultrasonic bath for 30 min. Next, the solution was diluted with water to a final volume of 10 mL and filtered using filter paper.

#### 2.5.2. Instruments and Quality Control

The following arsenic species: inorganic As(III), As(V) and dimethylarsenic (DMA) were determined using high performance liquid chromatography with hydride generation optical emission spectrometry detection (HPLC-HG-ICP-OES). The HPLC instrument was a liquid chromatograph (Shimadzu, Kyoto, Japan) with an anion-exchange column (Supelco, Bellefonte, PA, USA) LC-SAX1 (250 × 4.6 mm). The chromatographic elution was isocratic with a flow rate of 1 mL min^−1^ of phosphate buffer (5 mM Na_2_HPO_4_ and 50 mM KH_2_PO_4_ × 2H_2_O) and an injection volume of 200 µL. PEEK (polyetheretherketone) tubing was inserted into a Tygon sleeve for transfer of the eluent from the LC column to the in-spray chamber of the hydride generation unit (MSIS, Agilent, USA). The concentration of NaBH_4_ was 1% in 1% NaOH, the concentration of HCl was 5 M (all reagents Merck, Darmstadt, Germany). An inductively coupled plasma optical emission spectrometer Agilent 5110 ICP-OES (Agilent, USA) was used for signal detection with the parameters listed above. As an analytical signal, the peak height corrected by the noise level was used. The limits of quantification were 6, 18, 21 µg L^–1^ for As(III), DMA, and As(V), respectively. Due to a lack of certified reference materials for arsenic speciation in the analyzed matrix, the standard addition method was used for accuracy and traceability studies. Recovery at the level of 80–120% was found as satisfactory.

A detailed description of the equipment, reagents, analytical procedure and obtained metrological data has been presented in previous work [24].

### 2.6. Biochemical and Physiological Parameters of Plant Response to Substrate

Pigment content was assayed in methanolic extracts with colorimetric method [25]. Approximately 0.5 g of fresh leaf tissue was homogenized in 5 mL of 96% methanol using a mortar and a pestle and then centrifuged to obtain a clear supernatant (3600 rpm/ min,15 min). 0.05 mL of the extract was diluted with 0.95 mL of 90% methanol and the absorbance at λ = 470, 653 and 666 nm was measured using a Cary 300 Bio UV-Vis spectrophotometer. Chlorophyll a (Chl-a), b (Chl-b) and total carotenoid (Caro) contents were calculated using the following formulae:

Chl-a (mg L^−1^) = 15.65 A_666_ − 7.34 A_653_;

Chl-b (mg L^−1^) = 27.05 A_653_ − 11.21 A_666_;

Caro (mg L^−1^) = 1000 A_470_ − 2.86 Chl a − 129.2 Chl b/245.

Leaf samples of ~0.5 g were ground in liquid nitrogen and extracted with 6 mL of 80% methanol. Extracts were centrifuged (10,000 g, 20 min), and the obtained supernatant was used for the total phenolic content (TPC) and relative antioxidant activity assays.

Folin–Ciocalteu reagent was employed in order to determine the TPC in methanolic extracts [26]. The extracts (0.2 mL) were mixed with diluted (1:1 with water, v/v) Folin-Ciocalteu reagent (2 mL) and after 3 min 10% Na_2_CO_3_ (2 mL) was added. Following incubation in a dark (60 min, room temperature), the absorbance at λ = 765 nm was measured using a Varian Cary 300 Bio UV-Visible scanning spectrophotometer. The results were expressed as mg of gallic acid equivalents (GAE) per g of tissue fresh weight (mg GAE g^−1^ FW). The assay was performed in triplicate. 

The relative antioxidant activity of methanolic extracts was measured based on their scavenging activity towards a DPPH radical using a Cary 300 Bio UV-Vis spectrophotometer [27]. The reaction mixture comprised 0.5 mL of the extract, 3 mL of absolute ethanol and 0.3 mL of 0.5 mM DPPH ethanolic solution. After the incubation (100 min, in dark), the absorbance at λ = 517 nm was measured. Ethanol and the extract mixture was used as a blank. The reaction was performed in triplicates. The relative scavenging capacity (RSC) was calculated using the following formula:

$$RSC\;\left[\%\right]=\left\{\frac{Ac-Ae}{Ac}\right\}\times 100,$$

where: *A_c_* is the absorbance of the control; *A_e_* is the absorbance of the extract.

The contents of salicylic acid (SA) in leaves was determined with a HPLC-FLD method [28,29]. *Paulownia* leaves were ground in liquid nitrogen using a mortar and a pestle to obtain a fine powder and ~0.50 g was taken for analysis. SA was extracted twice with methanol (90% followed by a straight solvent) and each time sonicated for 15 min. After centrifugation (10,000 g, 20 min), the supernatants were combined, and the solvent was evaporated to dryness under a stream of nitrogen (industrial grade, ≥99.95% by Air Products, Poland). Dry extracts were redissolved in 3 mL of TCA (5%, w/v) for pigment precipitation and centrifuged (10,000 g, 10 min). SA was extracted from the aqueous phase three times with a mixture of ethyl acetate, cyclopentane and isopropanol (100:99:1, v/v/v). The organic phases were combined, and the solvent was evaporated to dryness under a stream of nitrogen. Dry extracts were stored at −24°C until analyses. Before analysis, dry residues were dissolved in the mobile phase (0.2 M KAc buffer, pH 5.0) and analyzed with a Waters Alliance 2695 Chromatograph coupled with a Waters 2475 Multi-λ Fluorescence Detector (Waters Corporation, Milford, MA, USA). The separations were performed using a Waters Spherisorb ODS2 column (100 × 4.6 mm, 3 μm) at a 1.5 mL min^−1^ flow rate. The fluorometric detection was performed at λ_Ex_ = 295 and λ_Em_ = 405 nm. SA was identified according to the retention time and quantified by comparing the peak area using an appropriate calibration curve.

Rhizosphere, root and leaf sample preparation for phenolic acids and low-molecular-weight organic acids (LMWAOs) analyses was carried out [30]. The samples were homogenized in 80% methanol and HCl (99:1), sonicated and shaken for 5 h. Extracts were dried and redissolved in water for organic acids and 80% methanol for phenolic acids. Aqueous and methanolic solutions were centrifuged (at 3600 rpm/ min for 15 min at 25 °C), and the resulting samples were filtered through 0.2 μm nylon filters prior to chromatographic analysis. For determination of phenolic compounds and organic acid a Waters Acquity H class UPLC system coupled with a Waters Photodiode Array Detector (Waters Corporation, Milford, MA, USA) and a Waters Acquity UPLC BEH C18 column (150 × 2.1 mm, 1.7 mm) were used. The gradient of water and acetonitrile (both containing 0.1% formic acid) was applied at a flow rate of 0.4 mL min^−1^. The quantification was conducted at λ = 280 and 320 nm for LMWOAs and phenolic compounds, respectively (Magdziak et al. 2020).

### 2.7. Statistical Analysis and Calculations

All statistical analyses were performed using STATISTICA 13.3 software (StatSoft, USA). To show the existence of uniform groups of objects (α = 0.05), the multiple comparison Tukey’s HSD test was performed following one-dimensional analysis of variance (ANOVA).

To define the effectiveness of element uptake by *Paulownia*, the bioconcentration factor (BCF) was calculated. It is the ratio of particular elements or their groups (NEs, REEs) concentration in plant organs (leaves, stem and root) to their concentration in soil. Additionally, to characterize the effectiveness of element transport from the root system to aerial plant parts, the translocation factor (TF) was calculated as the ratio of studied elements/groups of elements concentration in leaves and stem to their concentration in roots [31,32]. To perform a quantitative analysis of the element extraction ability of *Paulownia*, a Metal Extraction Ratio (MER) was calculated [33,34]. The MER value shows the suitability of the plants for phytoremediation [35], and is calculated according to the following formula:

MER (%) = {c_plant_ × m_plant_} / {c_soil_ × m_rootzone_} × 100,

where: c_plant_ is the element concentration in the harvested organs of the plant (stems and leaves), m_plant_ is the biomass of harvestable organs produced in one harvest, c_soil_ is the element concentration in the experimental system, and m_rootzone_ is the soil mass rooted by the plant during studies.

The plant effective number (PEN) was calculated to estimate, how many plants are needed to extract 1 g of determined elements from the substrate, taking into account the biomass of the above-ground organs [33,36].

## 3. Results

### 3.1. Substrates Properties

The control soil was characterized as sandy soil with a low content of total carbon and nitrogen (0.91 and 0.05%, respectively), slightly acidic (pH = 5.39), low salinity (EC = 0.27 mS) and low cation exchange capacity (CEC = 22.6 meq 100 g^−1^) (Table 1). Compared to the other variants, this soil was characterized by a content of the determined elements similar to geochemical background of Polish soils (Table 2).

The remaining substrates (FT, MS and their mixtures with BR) were characterized by an alkaline reaction with a pH ranging from 7.94 for MS/BR to 8.49 for FT/BR (Table 1), which resulted from alkali waste addition and was characterized by a pH of the aqueous solution of 10.1. Additionally, significantly higher salinity was noted for the MS and MS/BR substrates compared to the control soil (EC = 6.75 and 6.53 mS, respectively). Substrate pH and EC were directly related to a high sodium and calcium content. Sodium concentration in FT and MS was over 3 and nearly 5-fold higher than in control soil. Significantly higher calcium concentrations were also found in FT and MS samples compared to the control, i.e., ~22 and 19 times higher, respectively. The high concentration of these elements also influenced the base cation saturation ratio. MS and MS/BR substrates showed significantly higher concentrations of TC, SOC, SIC, N_t_, S_t_ compared to other variants (Table 1). The results obtained for MS most likely result from the presence of organic pollutants characteristic of this kind of waste, that disturb the correct estimation of the SOC value.

With only a few exceptions, the detected elements were present in significantly higher concentrations in FT, MS and their mixtures with BR compared to control soil (Table 2). Further, their concentration in MS was higher compared to FT except for Cs, K, Li, Mg, Mn and Sr. Extremely high concentrations of As, Cd, Pb, Zn were found in MS and MS/BR substrates and in the case of As it was ~1780 and 370 fold higher compared to the control for MS and FT, respectively. This metalloid was present mainly in inorganic forms in MS and MS/BR substrates, and the concentration of the most toxic As(III) in MS reached 1020 mg kg^−1^ and was ~165 fold higher than FT (Table 2).

Considering the particular fraction of elements determined by the BCR procedure, it was observed that for substrates FT and MS, most of analyzed elements was associated with the F1 fraction, which is the most soluble and most bioavailable for plants. For example, the F1 fraction for the MS substrate was associated with ~90%, 74% and 49% of total Cd, Cu and As, respectively. Biochar application for these substrates influenced metal speciation, especially for the F1 fraction. Biochar application led to a decrease in the F1 fraction for most analyzed elements (Table S1).

### 3.2. Paulownia Hybrid Growth

**Figure 1:**
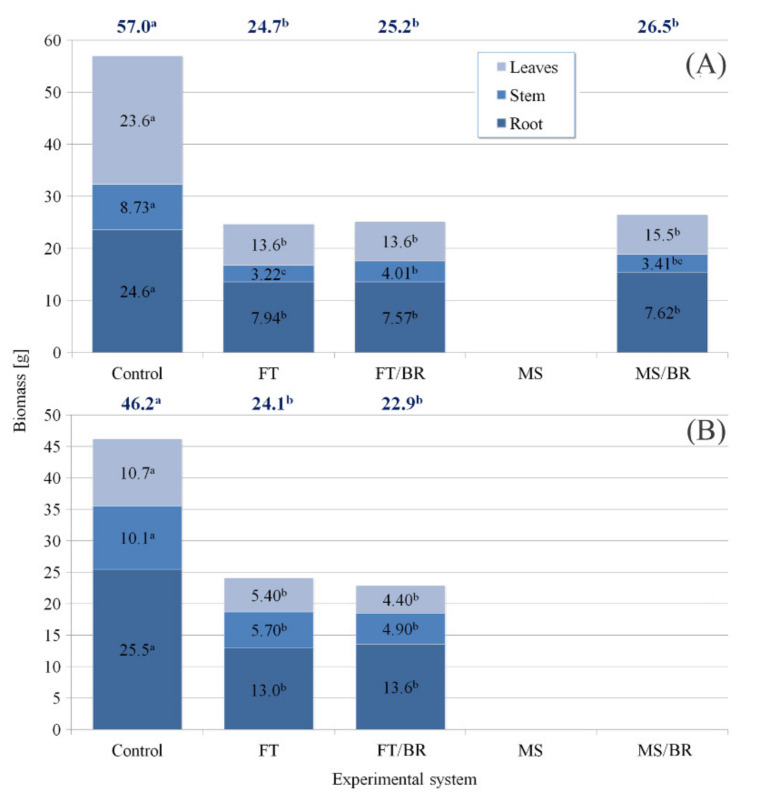
Characteristics of fresh biomass (g FW) of *Paulownia* growing under particular experimental systems in the first (**A**) and the second (**B**) year of investigation (identical lettering denote no significant (*p* > 0.05) differences according to a post-hoc Tukey’s HDS test).

The highest biomass in the first and the second year of the experiment was noted for control seedlings (57.0 and 46.2 g, respectively) (Figure 1). For FT, FT/BR and MS/BR substrates, significant inhibition of overall plant growth was observed with a mean biomass yield of 24.7, 25.2 and 26.5 g for FT, FT/BR and MS/BR in the first year and 24.1 and 22.9 g for FT and FT/BR in the second year. Seedlings cultivated in MS were unable to survive during the first year (Figure 1A) and also in MC/BR in the second year (Figure 1B) of the experiment. The biomass of particular organs of seedlings cultivated in waste materials (all variants) was similar and significantly lower compared to the control.

**Figure 2:**
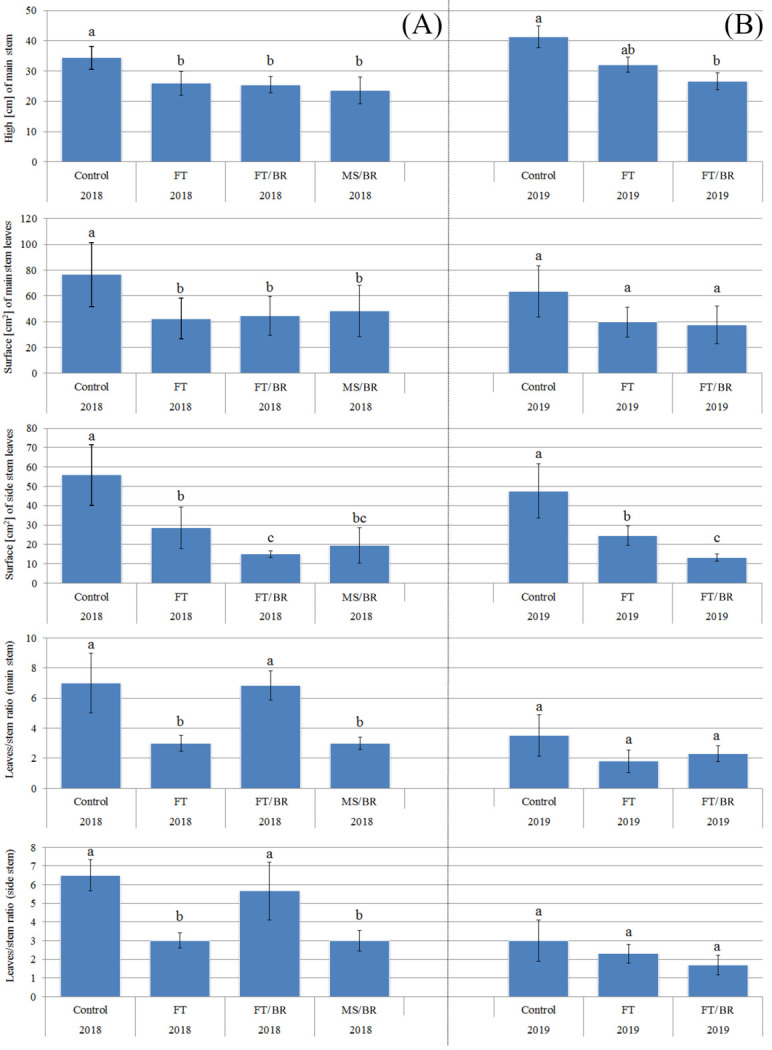
Characteristics of main stem height [cm], main and side stem leaves surface (cm^2^) and leaves/stem ratio of *Paulownia* cultivated in particular experimental systems in the first (**A**) and the second (**B**) year of investigation (identical lettering denote no significant (*p* > 0.05) differences according to a post-hoc Tukey’s HDS test).

In the first year, plant height reached 34.4 cm for the control and 25.9, 25.4 and 23.6 cm for the FT, FT/BR and MS/BR systems, respectively (Figure 2A). In the second year of cultivation, a significant difference in the biomass of the control and FT/BR seedlings was observed (41.2 and 26.6 cm, respectively) (Figure 2B). The surface of main and side stem leaves also reflected the inhibitory effect of the applied substrates (Figure 2A,B). Additionally, leaf/stem ratios both for main and side stems were significantly higher for the control and FT/BR than for FT and MS/BR plants in the first year of the experiment. Moreover, for these parameters, no significant differences were noted between the control, FT and FT/BR systems in the second year.

**Figure 3:**
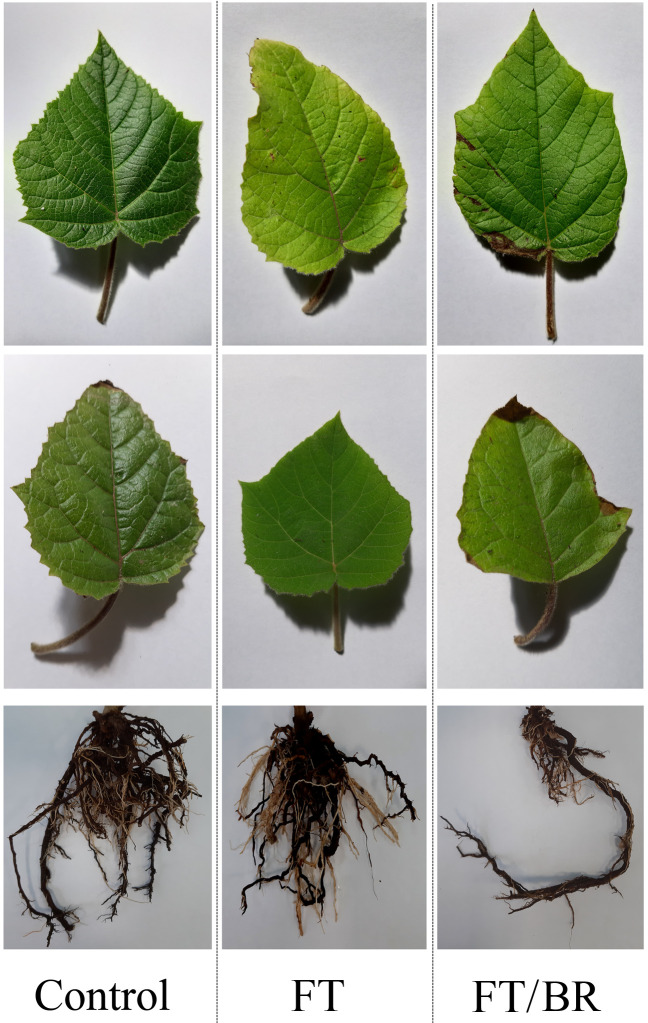
Leaves and roots of *Paulownia* after second year of the experiment.

In the first year of cultivation, plants suffered no visible leaf damage resulting from applied waste materials. In contrast, in the second year, leaf necrosis and chlorosis, along with root thinning, was observed for plants cultivated in FT/BR (Figure 3).

### 3.3. Element Uptake and Distribution in Paulownia Plants

Depending on the variant, a specific distribution of individual elements was found (Tables S2 and S3). After the first year of the experiment, elements were accumulated to a different extent in roots and shoots. At the same time, Ag, B, Ba, Bi, Ca, Co, Re and Ta were found in the aboveground organs exclusively. Plants cultivated in FT accumulated the majority of elements in roots or leaves, while their content in stems was significantly lower with the exception of Hg, Sn, Ta and Zn. The addition of BR altered the distribution of numerous elements in favor of their transport to the stem. The uptake of the majority of elements in plants cultivated in MS/BR was generally limited to the root system with effective transportation to the leaves observed only for particular elements (Ba, Bi, Hg, Mn, Si, Sn, Th, Tl, W and Zn).

In the second year of studies, both control plants and plants cultivated in flotation tailings (FT and FT/BR) were characterized by effective uptake to roots observed for the majority of elements, and to a different extent, to leaves. Effective accumulation in the stem was limited to selected elements, i.e., As, Bi, Cd, Co, Cs, Hg, Rb, Sn, Ta and W.

**Figure 4:**
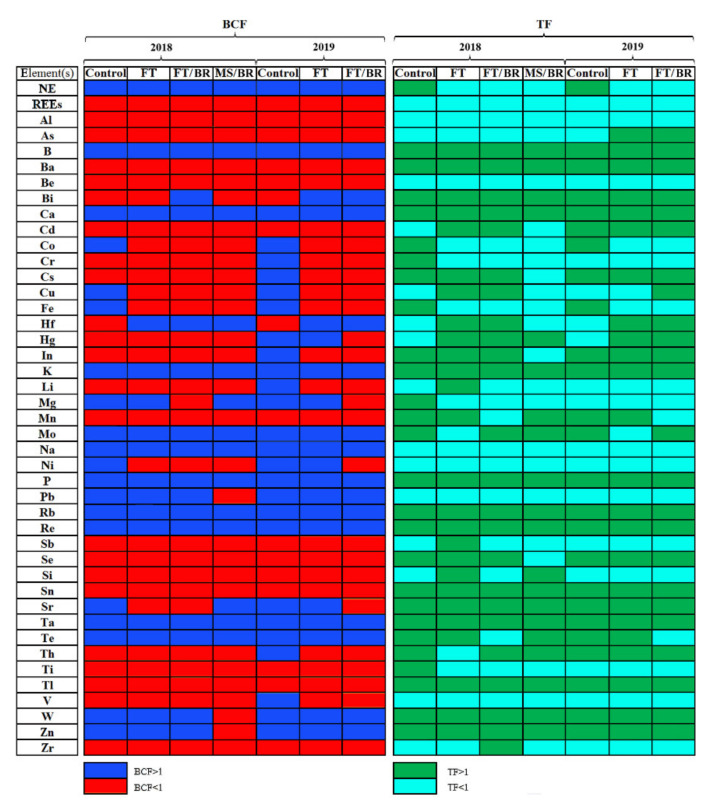
Bioconcentration factor (BCF) and translocation factor (TF) values calculated for determined elements in *Paulownia* cultivated in particular experimental systems.

BCF and TF values higher than 1 were calculated for B, Ca, K, P, Rb, Re and Ta (Figure 4). Effective uptake of NE, Hf, Mo, Na, Pb, Sr, Te, W and Zn was noted, and translocation for Ba, Bi, Cs, Hf, Hg, In, Mn, Mo, P, Rb, Sn, Sr, Te, W and Zn. Uptake and translocation of REEs (sum), Al, Be, and V were greatly limited compared to other elements.

Considering metal accumulation per plant, the highest content of the majority of elements was determined for MS/BR (2018), and in the case of B, Ca, Cu, In and Si, for the control (2018 and 2019). Further, despite significant differences in Be and Bi content in substrates, their accumulation was strongly inhibited and found to be at a similar level (Table 3).

In the second year of the study, the similarities and differences between the control plants and FT and FT/BR were less visible. Content of REEs and also Al, As, B, Cu, K, Mn, Na, Ni, Sb, Se, Tl, V, Zn and Zr were similar in the control and seedlings growing under FT. It was also observed that BR addition to FT substrate modified metal concentration on plant biomass. Compared to the FT substrate plants growing on the FT/BR substrate had a significantly lower concentration per plant of most of the analyzed elements. This trend was observed in both 2018 and 2019.

### 3.4. Quantitative Analysis of the Extraction Ability of Paulownia

Estimation of the uptake of particular elements using BCF or their transport from roots to shoots using TF does not explain the extraction ability of the studied *Paulownia* hybrid with respect to polluted substrates enriched or not with biochar. For this reason, MER values were calculated to show the % of elements removed from the control and especially polluted substrate during one harvest. Values higher than 0.001% and in at least one experimental system are shown in Table 4.

PEN calculations for individual elements showed that for the extraction of 1.0 g of a particular metal from the substrate a different number of plants was needed depending on the experimental system. The lowest PEN values were calculated for Ca, K, Mg, Na, P and Fe, which is a common observation due to the role of these metals in plant growth and development (Table 5).

The highest PEN values were observed for Be, Li and V. Promising values regarding practical aspects were noted for NE, Al, B, Ba, Cs, Cu, Mn, Pb, Si and Sr remediation, where several dozen to several hundred plants are needed to extract 1.0 g of the mentioned elements.

### 3.5. Pigment Content in Paulownia Leaves

Substrate modification did not cause any significant changes in chlorophyll content (Table S4). Simultaneously, increased chlorophyll b was observed for plants cultivated in mining sludge with the addition of biochar (MS/BR) compared to the control (~20%) and biochar supplemented flotation tailings (FT/BR) (~44%), and relatively lowered for FT/BR in comparison with pure FT (~20%). Carotenoid content increased for plants cultivated in waste materials, reaching the highest value for MS/BR (~180% compared to the control). Subsequently, a significant drop of the chlorophyll-to-carotenoid ratio versus control was noted, most pronounced for biochar enriched substrates (~32 and 47% for FT/BR and MS/BR, respectively).

### 3.6. Phenolic Metabolites in Paulownia Leaves and Roots

Substrate modification led to a significant decrease of the phenolic fraction in *Paulownia* leaves, clearly marked for plants cultivated in FT and MS/BR (by ~43 and 29%, respectively) (Table S4). Further, the addition of BR to FT resulted in a significant increase of total phenolic content compared to pure FT, although the value remained lower than in the leaves of the control plants. Relative changes in phenolic accumulation in leaves were closely reflected by total antioxidant capacity versus the DPPH radical (Table S4).

**Figure 5:**
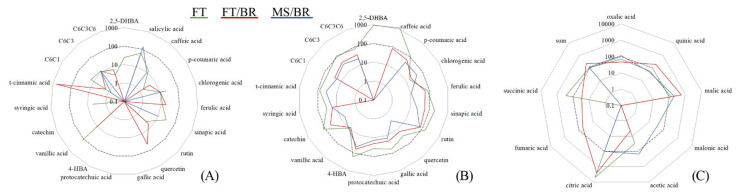
Percentage change of biochemical and physiological parameters of leaves (**A**) and roots (**B**,**C**) of *Paulownia* plants cultivated in waste materials (FT—flotation tailings, FT/BR—flotation tailings supplemented with biochar, MS/BR—mining sludge supplemented with biochar) in relation to control plants. For undetected compounds, the detection limit was used for calculations as a minimal detectable amount. Only significant differences were present at α = 95% (2,5-DHBA—2,5-dihydroxybenzoic acid, 4-HBA—4-hydroxybenzoic acid, C6-C1—hydroxybenzoic acids, C6-C3—phenylopropanoids, C6-C3-C6—flavonoids, sum—a sum of low-molecular-weight organic acids (LMWOAs)).

In the leaves and roots of control plants C6-C1 (hydroxybenzoic acids), C6-C3 (hydroxycinnamic acids/phenylpropanoids) and C6-C3-C6 (flavonoids) were assessed (Figure 5, Table S4).

In leaves, the dominant group was C6-C1 structures represented by 2,5-DHBA > 3,4-DHBA (protocatechuic) > 4-HBA > gallic acid. Other hydroxybenzoic acids (vanillic, syringic and salicylic) were present in smaller amounts. The sum of C6-C1 was ~3-fold higher than the C6-C3 and C6-C3-C6 phenolics, and among phenylpropanoids, ferulic acid was a dominant compound. Other hydroxycinnamic acids were detected in lower amounts (trans-cinnamic, caffeic, *p*-coumaric, chlorogenic, ferulic, sinapic acids). Among flavonoids, rutin, quercetin and catechin were detected at relatively lower levels.

The use of waste materials significantly affected the metabolic profile in leaves, and a strong inhibition of phenolics accumulation was clearly noted (Figure 5). Among the C6-C1 compounds, 2,5-DHBA, salicylic and syringic acids were detected in significantly lower amounts, while gallic, protocatechuic and 4-HBA were not detected in the case of the FT substrate. The C6-C3 acids were accumulated in significantly lower amounts, while trans-cinnamic acid was not detected, and only rutin was detected among flavonoids. For the FT/BR substrate, an overall suppression of C6-C1 acids, caffeic, sinapic acids and catechin was observed. The strongest inhibition of phenolic accumulation was noted for MS/BR, with the exception of salicylic acid, for which a ~30% increase was observed in comparison with the control. Considering C6-C3 structures, only a few acids were quantified at lower levels, i.e., caffeic, chlorogenic and sinapic acids.

In roots, the dominant group was also C6-C1, with gallic and vanillic acids as the main compounds, while quercetin was a major flavonoid (Table S4). Similarly to leaves, the addition of waste material led to quantitative changes in the phenolic profile. For FT substrate, an increase in 2,5-DHBA, 4-HBA, caffeic, *p*-coumaric and sinapic acids, rutin and catechin content was observed, while the synthesis of other compounds was inhibited compared to the control (Figure 5). In contrast, the FT/BR substrate decreased the content of phenolic compounds in comparison to the control and 2,5-DHBA and trans-cinnamic acid were not detected. Also, for the MC/BR group a suppressed synthesis of phenolics was observed, and 2,5-DHBA and caffeic acid were not present. Contrary to leaf tissue, the relative changes in C6-C1, C6-C3, C6-C3-C6 content in roots shared a similar pattern with the exception of 2,5-DHBA, caffeic and *p*-coumaric acids (Figure 5).

### 3.7. LMWOAs in Paulownia Roots

Considering all experimental groups, eight LMWOAs were detected in the roots of *Paulownia* (Table S4). Roots of the control plants contained seven from eight of the determined acids, and malic and acetic acids were dominant. The substrate modification significantly affected the profile of organic acids (Figure 5). For FT substrate, malic and succinic acids were dominant and a significant increase of succinic acids relative to the control was noted (~270%). In the roots of FT/BR plants, a significant increase of malic acid was observed compared to the control and FT plants (~560 and 470%, respectively).

In contrast, the accumulation of oxalic, fumaric and succinic acids was significantly decreased compared to control and FT-cultivated plants (Figure 5). Further, citric acid was detected only in the roots of plants cultivated in FT and FT/BR substrates at comparable levels (Table S4). In the case of MS/BR substrate, malic and acetic acid were dominant, and the sum of LMWOAs was assessed at a comparable level to the control plants.

## 4. Discussion

### 4.1. Biomass Yield and Phytoextraction Efficiency of Paulownia Hybrid

Generally, *Paulownia*, especially *P. tomentosa* prefers high acidity, although the best growth is obtained at strongly acidic to mildly alkaline pH [37,38]. In general, the bioavailability of elements is higher under acidic soil conditions and decreases with increasing pH [39]. Under alkaline conditions, the mobility of some elements decreases, mainly due to the ability of Ca compounds to absorb them [40]. In the present experiment, the control soil reaction was acidic. At the same time, applied waste materials showed an alkaline reaction with a pH range from 7.94 to 8.49 for MS/BR and FT/BR, respectively. The efficiency of phytoextraction depends on many factors, among which the properties of the substrate play an important role. The worst growth conditions for plants were determined for the MS and MS/BR substrates, which resulted not only from the extreme content of the analyzed elements, but also from their poor physicochemical parameters (Table 1 and Table 2). The physical properties of these substrates, mainly determined by granulometric composition, can fundamentally affect the availability of both nutrients and toxic elements [41]. Water retention and water availability for plants also depend on the physical properties of substrates which can directly determine the bioavailability of elements. In normal soil conditions, fine particles usually show a higher water retention capacity, but in the case of the substrates used in the experiment, especially the MS substrate, the predominance of fine sized particles cause disturbed water infiltration, limited by the poor structural properties of this kind of waste [42]. The predominance of fine fractions in these materials, and especially in MS, leads to an increase in their density, which inhibits penetration by plant roots, significantly hindering their proper growth and the ability to take up elements.

Moreover, excessive compaction of these wastes can also result in a disturbance of air conditions, which in turn cause significant obstruction or inhibition of growth. In poor ventilation conditions, plants may show a deficiency of nutrients, even with their high concentration in the substrate. Additionally, more toxic elements like As, Pb and Cd are strongly associated with fine fractions, which can also influence their bioavailability [43]. In the case of this type of waste, the application of porous materials, such as biochar, may contribute to a significant loosening of their structure and an improvement of air-water properties, which directly affect the efficiency of phytoextraction processes. The use of biochar due to the small specific surface area (50.1 m^2^ g^−1^) aimed to increase the chances of plants adapting to new, unfavorable growth conditions. Adsorption on biochar particles because of the alkaline pH (10.0) was a factor limiting the rapid increase in the concentration of toxic elements in the rhizosphere and ultimately facilitating the growth of seedlings. The use of biochar with respect to active carbons seems to be more justified because it is not about highly effective sorption but limiting the sudden increase in the concentration of metals negatively affecting plant growth, especially at the beginning of the phytoextraction process.

However, in our study, biochar did not significantly affect the investigated physicochemical properties, disregarding the bioavailable forms of selected elements in the F1 fraction, which decreased in FT/BR and MS/BR substrates compared to pure waste materials. The reduction of the F1 fraction of the analyzed elements resulted in a decrease in the content of the analyzed elements in the *Paulownia* biomass, which was clearly observed when comparing the FT and FT /BR substrate both in 2018 and 2019. Most of the analyzed elements were significantly lower in the biomass of plants growing on FT / BR substrate compared to the FT substrate, which clearly indicates the influence of biochar on the reduction of the bioavailability of elements. This fact may suggest that even a small addition of biochar with a small specific surface may significantly affect the behavior of *Paulownia*.

The high efficiency of metal phytoextraction by *Paulownia* proved its high resistance to elevated concentrations of toxic elements in cultivation substrates such as mining waste materials. However, a drop in temperature below 0 °C for several days in January 2019 could have caused an aggravation of substrate-derived stress and consequently led to the death of plants cultivated in the most polluted MS/BR. Moreover, seedlings exposed to MS were unable to grow, and a possible cause of their withering could be an extremely high concentration of DMA (42.4 mg kg^−1^) which has previously proved highly toxic to woody plants [44,45]. The breakpoint of DMA concentration in modified Knop solution described in the above mentioned papers was 41.4 mg kg^−1^, while higher concentrations resulted in damage to young tree seedlings. This could explain why seedlings exposed to MS/BR with a DMA concentration of 8.06 mg kg^−1^ were able to grow.

Due to the effect of the chemical composition of the substrates, it is difficult to clearly state to what extent the results obtained in this work rank the studied hybrid in a hierarchy based on the potential of this species for effective metal phytoextraction. Oxytree could be an effective and selective accumulator of NE, B, Hf, K, Mg, Mo, Na, P, Pb, Rb, Re, Sr, Ta, Te, W and Zn as confirmed by a BCF > 1. Moreover, TF > 1 calculated for Ba, Be, Bi, Cd, Cs, Cu, Hf, Hg, In, K, Mn, Mo, P, Rb, Re, Se, Sn, Sr, Ta, Te, Th, Tl, W and Zn directly demonstrates the significant potential of this hybrid for the remediation of highly contaminated wastes. Unfortunately, calculated MER values showed that extraction of the studied elements was limited. All values of MER were lower than 1%, which is typical for the majority of plants and suggests that treatment of the waste used in the experiment will require the growth of *Paulownia* over several hundreds of years [33]. On the other hand, MER values calculated for selected elements (B, Cu, Sr or Ta) in control plants in the first or the second year of the experiment suggest that this process could be significantly shortened. It should also be emphasized that the calculations performed relate to one plant. Therefore, in the case of the practical use of the tested hybrid in the form of a larger number of individuals, there may be an additional factor shortening the time of the remediation. This is where the question of the number of plants is justified. For this reason, the PEN was calculated for individual elements and their groups (NE and REEs). To extract 1 g of elements such as NE, Al, B, Ba, Cs, Cu, Mn, Pb, Si and Sr from the substrate, the number of plants (shoots) was lower than 2500, which confirms the potential of studied *Paulownia* hybrid to remediate substrate polluted with selected elements only. PEN values calculated previously for Cd in six Chinese cabbage cultivars were from 3745 to 53,433 [33], while for *Paulownia* from 5800 (plants growing under MS/BR system) to 455,000 plants (control in the first year of the study). On the other hand, PEN values for Pb and Zn were 351–3252 and 220–1279 shoots, respectively, while in the case of *Piptatherum miliaceum* (L.) Cosson they were 22,000 and 4300 shoots, respectively [36]. It should be remembered that PEN values are often calculated for whole plants, also taking into account the root that effectively accumulates metals. This, of course, significantly reduces the number of plants needed for effective remediation, although in our opinion, providing such results would be quite debatable due to the possibility of growth of the studied *Paulownia* hybrid for many years.

The limited uptake of selected elements may be the effect of the high pH of the applied substrates, which may be the next factor able to increase MER values. However, limited translocation and metal accumulation in roots have previously been described for *Paulownia* [14]. The authors studied two *Paulownia* lines of different leaf sizes, showing varying efficiency in Pb and Zn phytoextraction. This suggests that the accumulation of metals may be closely related to the size of plants. Any change in soil pH strongly affects metal solubility and its bioavailability for plants [46]. In alkaline reaction, element mobility generally decreases; this is usually associated with an extensive calcium level. Excessive levels of calcium, especially in MS substrate can also affect nutrient (Mg, P, Mn, Fe) bioavailability and uptake, which can explain the low content of this element in the whole biomass of *Paulownia* compared to the control, especially for the FT and FT/BR substrates, as well as the significant reduction of biomass. Under normal conditions, Ca is a dominant component among base cation in the soil sorption complex and plays an extremely important role, both in maintaining soil fertility and proper plant functioning. In our study Ca^2+^ concentration in the sorption complex of FT and MS substrates was over 70 and 55 fold higher respectively, compared to the control. Despite the positive role played by this cation, in such extremely high concentrations, it can disturb the proper management of other plant nutrients present in the soil solution, such as K^+^ and Mg^2+^ [47]. An important parameter modifying plant growth conditions is the salinity of the substrate caused by excessive salt concentration, including heavy metal salts. On the MS and MS/BR substrates, the electrolytic conductivity (EC) was 6.75 and 6.53 mS cm^−1^ respectively, which was much higher in comparison with the other experimental substrates. In such conditions, plants have a difficult uptake of water from the substrate, and as a result of osmotic stress, their growth slows down [48]. This is additionally enhanced by the predominance of the fine fraction in these materials.

### 4.2. Physiological Response

*Paulownia*, like other plants, has a characteristic physiological response [49,50,51]. The photosynthetic activity and physiological state of *Paulownia* plants were indirectly measured by assessing pigment content in leaves [52]. However, waste materials did not cause any negative changes in chlorophyll content despite triggering severe growth retardation. The increase of carotenoid content without significant changes in the amount of chlorophyll was manifested in a slightly decreased chlorophyll-to-carotenoid ratio. This may indicate enhanced antioxidant activity to compensate for the inhibition of phenolic biosynthesis showing high ROS-scavenging properties. During metal stress, the action of lipophilic carotenoid is most likely targeted at the stabilization of membrane lipids from hydrophilic oxidizers [53].

Phenolic compounds are secondary metabolites, playing numerous functions in plant metabolism, including defense mechanisms against various stressors. They are recognized as biomarkers of plant response in conditions of elevated metal concentration in soil/substrate [54,55], and their enhanced biosynthesis under metal stress, along with their antioxidant action, has been confirmed for many plants and various tissue types [45,55,56,57,58]. Surprisingly, in the present study the accumulation of phenolic metabolites was noted neither in leaves nor roots of *Paulownia elongata S. × Paulownia fortunei*. The obtained results clearly indicated that TPC and the biosynthesis of individual metabolites were inhibited, as proved by the decreased total antioxidant activity values. In addition, hydroxybenzoic acid biosynthesis was more susceptible to inhibition than hydroxybenzoic acids, especially in leaves. In defense mechanisms under metal stress, phenolic compounds play the role of electron donors in the ROS scavenging system. However, even phenolic acids with high antioxidant potentials such as caffeic, chlorogenic and ferulic were not accumulated in *Paulownia* leaves and roots. The oxidative mechanisms resulting from metal stress are represented by both cinnamic and benzoic phenolic acids by either chelating heavy metals or removing free radicals [59]. However, these mechanisms were not observed in *Paulownia* cultivated in waste materials. Considering the phenolic profile, breaks of the metabolic pathway occurred at different stages depending on the waste material used, which is a start point for future studies.

Plant stressors also activate other intercellular defense mechanisms in which low-molecular-weight organic acids are involved. Their role is to maintain the homeostasis of elements in cells [60], but they also participate in main metabolic processes such as photosynthesis, respiration and amino acid biosynthesis. However, their most important role is the transport of cations and the detoxification of toxic elements (metals/metalloids) [30,61]. Thus, the presence of organic acids is a direct reflection of the physiological state of plants under stress from the environment, including substrate properties [62].

In the present study, the sum of detected LMWOAs in *Paulownia* roots was similar for the control and plants cultivated in FT, which was characterized by a high content of K, Ca and Mg. However, a significant decrease of oxalic and acetic acids content was observed, with a simultaneous increase in malic and citric acids. For biochar supplemented FT, the sum of detected acids was more than two-fold higher, with preservation of the tendency mentioned above. The observed changes may be related to the substrate characteristics, where the soluble cations, predominantly Ca and Mg, may have been exchanged with non-exchangeable and buffered soluble K as well as by organic acids produced by plant roots into the rhizosphere [63]. Moreover, plants have developed several means of metal detoxification, such as separation, chelation, avoidance and exclusion, which involve LMWOAs via extra- and/or intracellular mechanisms [64,65]. Many studies are in accordance with our results and have reported that plants are able to accumulate significant amounts of heavy metals such as Cd, Cu, Pb and Zn, transfer them to the aerial parts and store them in vacuoles as complexes with citric and malic ligands [66]. Increased biosynthesis of LMWOAs under the FT/BR system in *Paulownia* roots could also be related to the application of highly porous materials, such as biochar, which caused a significant loosening of substrate structure and improvement of air-water properties, thus directly affecting the defense mechanisms [60]. In the case of MS-cultivated *Paulownia* plants, the accumulation of LMWOAs in roots decreased, while the addition of BR to MS again stimulated their synthesis. The significant decrease in acid content may be related to the elemental overload in the substrate, especially of Cu, Cd, Zn and Pb, with a concomitant decrease of plant biomass. However, it is indisputable that increasing soil loosening with BR again stimulated acid synthesis.

### 4.3. Paulownia as an Effective but Invasive Remediator

The expansion of invasive (alien) species is a significant ecological problem for a number of reasons, including the mixing of species ranges and ultimately the homogenization of the environment. According to the Bern Convention on the Conservation of European Wildlife and Natural Habitats, it is necessary to protect species and habitats through legislative and administrative measures [67]. For this reason, the practical use of invasive species such as *Paulownia*, citing the Opinion of the Committee for Plants of the State Council for Nature Conservation [68], for remediative purposes seems to be unreasonable.

*Paulownia* prefers higher temperatures and significant water requirements. Therefore the first should be a proper selection of clones [69]. Clones with a greater resistance to lower temperatures may be of particular importance in terms of their viability. This should be considered in view of the progressive migration of invasive species due to climate change, which may lead to a gradual shift of the current range. This phenomenon may also be accompanied by the displacement of native species of trees and shrubs [70]. The selection of clones capable of effective phytoextraction of toxic metals, but most of all characterized by high resistance and currently considered invasive species, requires the implementation of specific procedures to limit their negative impact on the native fauna. These climatic changes may, in the future, limit the regrowth of native and valued species and promote those invasive species that are more responsive to growth in arid, polluted or dry areas [71,72].

## 5. Conclusions

*Paulownia* showed a varying ability to uptake and translocate individual metals, depending on the substrate on which it grew. The reduction of biomass compared to the control plants was an obvious consequence of the presence of waste in the substrate. On the other hand, the addition of biochar to highly polluted mining sludge suggests its important role in increasing the adaptation of this plant to unfavorable growth conditions. The obtained results suggest that growth on wastes with moderate concentrations of metals could limit the physiological response of *Paulownia* and thus facilitate its growth in subsequent years of the phytoextraction process.

The present studies clearly demonstrated that LMWOAs biosynthesis in plant roots plays an important role in adaptation, tolerance and detoxification mechanisms under conditions of excessive concentrations of toxic elements. Therefore, acid profiling may be a key tool to elucidate the nature of plant acclimation and may ultimately aid the development of new plant varieties that are tolerant to environmental changes. Thus, plant metabolomics should be widely employed to evaluate stress biology by identifying different compounds produced in response to environmental stressors and the role these compounds play in acclimation or tolerance responses. Phenolic compounds play an important role in response to metal stress in plants, which occurs as an increase in their overall content or enhanced biosynthesis of particular metabolites. However, the observed depletion in phenolic biosynthesis in *Paulownia* plants cultivated in heavily polluted waste materials does not illustrate their protective role in defense and adaptation mechanisms and this phenomenon requires further research.

## Figures and Tables

**Table 1:** Characteristics of experimental substrates.

Parameter		Control	FT	FT/BR	MS	MS/BR
Granulometric composition of soil	sand 2–0.05 %	84	34	39	1	7
	silt 0.05–0.02 (%)	14	60	54	89	85
	clay < 0.002 (%)	2	6	7	10	8
	granulometric fraction	S	SiL	SiL	Si	Si
pH	H_2_O	5.39 ^e^ ± 0.34	8.30 ^b^ ± 0.04	8.49 ^a^ ± 0.01	8.19 ^c^ ± 0.04	7.94 ^d^ ± 0.02
EC	mS	0.27 ^d^ ± 0.01	0.44 ^c^ ± 0.01	0.51 ^e^ ± 0.04	6.75 ^a^ ± 0.16	6.53 ^b^ ± 0.06
CEC	meq/100g	22.6 ^c^ ± 4.01	186 ^b^ ± 6.9	188 ^ab^ ±12.5	198 ^ab^ ± 5.74	201 ^a^ ± 4.28
Base cations (meq/100g)	Ca^2+^	2.60 ^c^ ± 0.03	181 ^a^ ± 6.66	182 ^a^ ± 12.5	142 ^b^ ± 5.13	144 ^b^ ± 3.25
	Mg^2+^	0.29 ^c^ ± 0.02	4.15 ^b^ ± 0.16	4.26 ^b^ ± 0.05	43.2 ^a^ ± 0.53	43.9 ^a^ ± 1.04
	K^+^	0.04 ^d^ ± 0.00	0.82 ^c^ ± 0.01	0.84 ^c^ ± 0.02	3.63 ^a^ ± 0.04	3.42 ^b^ ± 0.04
	Na^+^	0.32 ^d^ ± 0.01	0.54 ^c^ ± 0.04	0.74 ^b^ ± 0.03	9.62 ^a^ ± 0.11	9.54 ^a^ ± 0.11
Base cations saturation (%)	Ca^2+^	11.8 ^c^ ± 2.38	97.0 ^a^ ± 0.02	96.9 ^a^ ± 0.16	71.5 ^b^ ± 0.53	71.6 ^b^ ± 0.52
	Mg^2+^	1.34 ^c^ ± 0.36	2.23 ^b^ ± 0.01	2.27 ^b^ ± 0.14	21.8 ^a^ ± 0.42	21.9 ^a^ ± 0.42
	K^+^	0.20 ^d^ ± 0.04	0.44 ^c^ ± 0.02	0.45 ^c^ ± 0.04	1.83 ^a^ ± 0.03	1.70 ^b^ ± 0.04
	Na^+^	1.42 ^b^ ± 0.30	0.29 ^c^ ± 0.01	0.39 ^c^ ± 0.05	4.86 ^a^ ± 0.10	4.76 ^a^ ± 0.15
	Total	14.8 ^b^ ± 3.05	100 ^a^ ± 0.00	100 ^a^ ± 0.00	100 ^a^ ± 0.00	100 ^a^ ± 0.00
Bio-P	mg P_2_O_5_/100 g	37.9 ^a^ ± 1.61	4.26 ^d^ ± 2.11	2.73 ^d^ ± 1.04	19.7 ^b^ ± 1.55	14.4 ^c^ ± 1.78
Bio-K	mg K_2_O/100 g	1.98 ^c^ ± 0.06	20.5 ^b^ ± 0.59	20.7 ^b^ ± 0.33	119 ^a^ ± 2.53	116 ^a^ ± 0.62
ST	%	0.01 ^c^ ± 0.00	0.09 ^b^ ± 0.01	0.10 ^b^ ± 0.00	1.86 ^a^ ± 0.26	1.92 ^a^ ± 0.04
TN		0.05 ^b^ ± 0.01	0.03 ^bc^ ± 0.01	0.03 ^c^ ± 0.01	0.25 ^a^ ± 0.01	0.26 ^a^ ± 0.01
TC		0.91 ^c^ ± 0.08	5.97 ^b^ ± 1.13	5.82 ^b^ ± 0.22	11.4 ^a^ ± 0.16	11.9 ^a^ ± 0.34
TOC		0.80 ^d^ ± 0.04	1.01 ^c^ ± 0.14	1.41 ^b^ ± 0.53	4.27 ^a^ ± 0.06	4.28 ^a^ ± 0.05
SIC		0.10 ^d^ ± 0.04	4.96 ^b^ ± 1.00	4.41 ^d^ ± 0.37	7.13 ^a^ ± 0.18	7.62 ^a^ ± 0.28
TOC/TN		16.1 ^c^ ± 2.90	30.5 ^b^ ±1.96	41.9 ^a^ ± 8.96	17.3 ^c^ ± 0.46	16.5 ^c^ ± 3.18
C_HA_		0.31 ^a^ ± 0.02	0.11 ^b^ ± 0.01	0.14 ^b^ ± 0.01	0.32 ^a^ ± 0.03	0.30 ^a^ ± 0.05
C_FA_		0.20 ^a^ ± 0.07	0.04 ^bc^ ± 0.01	0.04 ^c^ ± 0.00	0.05 ^b^ ± 0.01	0.04 ^c^ ± 0.00
C _HA_/C_FA_		1.72 ^c^ ± 0.57	2.82 ^c^ ± 0.53	3.94 ^b^ ± 1.66	6.54 ^a^ ± 1.86	7.50 ^a^ ± 1.25
C_Humins_		0.29 ^d^ ± 0.09	0.79 ^c^ ± 0.04	1.75 ^b^ ± 0.05	3.90 ^a^ ± 0.18	4.14 ^a^ ± 1.06

Mean values (*n* = 3) ± standard deviations. Identical superscripts denote no significant (*p* > 0.05) differences according to a post-hoc Tukey’s HDS test; EC—electrical conductivity, S—sand, LS—loamy sand, SL—sandy loam, SiL—silt loam, Si—silt; Bio-P—bioavailable phosphorus, Bio-K—bioavailable potassium, ST—total sulfur, TN—total nitrogen, CEC—cation-exchange capacity, TC—total carbon, TOC—total organic carbon, SOC—soil organic carbon, SIC—soil inorganic carbon, C_HA_—carbon of humic acids, C_FA_—carbon of fulvic acids, C_Humins_—carbon of humins.

**Table 2:** Concentration of major and trace elements (mg kg ^−1^ DW) in substrates used in the experiment (*n* = 3).

Element(s)	Control	FT	FT/BR	MS	MS/BR
Ca	1240 ^c^	26,900 ^a^	26,100 ^a^	23,300 ^b^	22,700 ^c^
K	279 ^c^	7170 ^a^	6840 ^a^	3160 ^b^	2950 ^b^
Mg	188 ^c^	4190 ^a^	4020 ^a^	3210 ^b^	3220 ^b^
Na	118 ^c^	383 ^b^	365 ^b^	566 ^a^	476 ^a^
P	438 ^a^	350 ^b^	346 ^b^	439 ^a^	405 ^a^
Al	2217 ^c^	18,100 ^a^	17,700 ^a^	9220 ^b^	9130 ^b^
As	6.46 ^c^	31.1 ^b^	25.5 ^b^	11,500 ^a^	11,100 ^a^
As(III)	bDL	6.18 ^b^	5.82 ^b^	1020 ^a^	987 ^a^
As(V)	bDL	4.98 ^b^	4.52 ^b^	10,290 ^a^	9970 ^a^
DMA	bDL	bDL	bDL	42.4 ^a^	8.06 ^b^
As_org_	bDL	19.9 ^b^	15.2 ^b^	150 ^a^	135 ^a^
B	1.60 ^c^	4.48 ^b^	3.00 ^bc^	117 ^a^	110 ^a^
Ba	92.0 ^c^	564 ^b^	543 ^b^	4750 ^a^	4600 ^a^
Be	1.29 ^c^	1.84 ^a^	1.65 ^b^	1.47 ^bc^	1.31 ^c^
Bi	1.67 ^b^	1.47 ^b^	1.41 ^b^	5.62 ^a^	5.38 ^a^
Cd	1.31 ^b^	1.54 ^b^	1.35 ^b^	1730 ^a^	1670 ^a^
Co	1.30 ^c^	8.65 ^b^	8.23 ^b^	102 ^a^	95.9 ^a^
Cr	4.26 ^c^	21.5 ^b^	21.4 ^b^	619 ^a^	616 ^a^
Cs	157 ^c^	933 ^a^	901 ^a^	586 ^b^	528 ^b^
Cu	3.18 ^c^	5009 ^b^	4780 ^b^	7870 ^a^	7710 ^a^
Fe	2520 ^c^	7550 ^b^	7290 ^b^	27,900 ^a^	27,300 ^a^
Hf	1.35 ^a^	1.51 ^a^	1.49 ^a^	2.00 ^a^	1.92 ^a^
Hg	1.54 ^b^	1.90 ^b^	1.64 ^b^	70.9 ^a^	67.3 ^a^
In	2.98 ^a^	4.05 ^a^	3.16 ^a^	5.95 ^a^	5.26 ^a^
Li	2.50 ^c^	24.6 ^a^	23.9 ^a^	19.6 ^b^	17.8 ^b^
Mn	116 ^d^	970 ^a^	873 ^ab^	731 ^bc^	697 ^c^
Mo	1.89 ^c^	6.61 ^b^	3.75 ^bc^	19.4 ^a^	18.2 ^a^
Ni	2.23 ^c^	14.1 ^b^	12.4 ^b^	572 ^a^	496 ^a^
Pb	17.1 ^c^	56.8 ^b^	53.4 ^b^	1666 ^a^	1646 ^a^
Rb	3.23 ^c^	42.4 ^a^	37.8 ^a^	18.2 ^b^	18.1 ^b^
Re	1.48 ^a^	1.75 ^a^	1.56 ^a^	2.02 ^a^	1.64 ^a^
Sb	4.37 ^b^	4.85 ^b^	3.88 ^b^	229 ^a^	207 ^a^
Se	20.3 ^c^	25.6 ^b^	22.5 ^b^	179 ^a^	171 ^a^
Si	348 ^c^	819 ^ab^	743 ^b^	850 ^a^	723 ^b^
Sn	2.08 ^c^	31.7 ^b^	22.5 ^b^	179 ^a^	171 ^a^
Sr	4.74 ^c^	361 ^a^	340 ^a^	193 ^b^	152 ^b^
Ta	1.31 ^b^	1.59 ^b^	1.49 ^b^	4.06 ^a^	3.83 ^a^
Te	5.15 ^c^	9.15 ^b^	6.71 ^bc^	21.4 ^a^	19.9 ^a^
Th	3.48 ^c^	12.7 ^b^	12.2 ^b^	31.0 ^a^	32.6 ^a^
Ti	239 ^c^	350 ^a^	316 ^b^	334 ^a^	322 ^ab^
Tl	3.27 ^b^	4.16 ^b^	4.00 ^b^	191 ^a^	182 ^a^
V	7.37 ^c^	25.5 ^b^	23.7 ^b^	123 ^a^	122 ^a^
W	3.00 ^b^	3.58 ^b^	3.15 ^b^	1230 ^a^	1220 ^a^
Zn	14.2 ^c^	53.7 ^b^	52.9 ^b^	12,500 ^a^	12,300 ^a^
Zr	4.08 ^c^	8.66 ^b^	7.09 ^b^	65.7 ^a^	63.3 ^a^
NE	49.8 ^c^	114 ^b^	76.2 ^bc^	808 ^a^	535 ^b^
REEs	32.5 ^c^	82.9 ^a^	72.1 ^ab^	76.4 ^a^	55.8 ^b^

Identical superscripts denote no significant (*p* > 0.05) differences between elements content in particular substrates (within a rows) according to a post-hoc Tukey’s HDS test; bDL—below detection limit.

**Table 3:** Content of major and trace elements (mg per plant DW) in *Paulownia* plants growing in particular experimental systems (*n* = 3).

Element(s)	2018				2019		
	Control	FT	FT/BR	MS/BR	Control	FT	FT/BR
Ca	652 ^a^	412 ^b^	320 ^b^	610 ^a^	585 ^a^	442 ^b^	270 ^c^
K	241 ^b^	245 ^b^	209 ^c^	283 ^a^	272 ^a^	264 ^a^	172 ^b^
Mg	66.6 ^b^	43.3 ^c^	21.0 ^d^	92.2 ^a^	104 ^a^	49.6 ^b^	22.1 ^c^
Na	38.6 ^b^	39.2 ^b^	40.9 ^b^	54.7 ^a^	55.5 ^a^	59.8 ^a^	43.3 ^a^
P	131 ^a^	7.10 ^b^	7.04 ^b^	12.3 ^b^	97.6 ^a^	12.3 ^b^	7.06 ^b^
Al	18.9 ^ab^	17.3 ^ab^	10.8 ^b^	58.8 ^a^	21.7 ^a^	21.3 ^a^	13.3 ^b^
As	0.08 ^b^	0.10 ^b^	0.05 ^b^	1.42 ^a^	0.12 ^a^	0.13 ^a^	0.06 ^b^
B	1.40 ^a^	0.47 ^b^	0.35 ^c^	1.85 ^a^	0.72 ^a^	0.46 ^b^	0.28 ^c^
Ba	0.62 ^d^	0.96 ^b^	0.79 ^c^	1.30 ^a^	0.78 ^b^	1.21 ^a^	0.73 ^b^
Be	<0.0 ^a^	<0.0 ^a^	<0.0 ^a^	<0.0 ^a^	<0.0 ^a^	<0.0 ^a^	<0.01 ^a^
Bi	0.01 ^a^	0.01 ^a^	0.01 ^a^	0.01 ^a^	0.01 ^a^	0.02 ^a^	0.01 ^a^
Cd	<0.0 ^c^	0.01 ^b^	<0.01 ^c^	1.03 ^a^	0.01 ^b^	0.01 ^a^	<0.01 ^b^
Co	0.02 ^c^	0.05 ^b^	0.03 ^c^	0.12 ^a^	0.04 ^b^	0.08 ^a^	0.04 ^b^
Cr	0.06 ^b^	0.04 ^c^	0.04 ^c^	0.11 ^a^	0.09 ^a^	0.06 ^b^	0.04 ^b^
Cs	2.72 ^b^	1.60 ^c^	1.26 ^d^	3.18 ^a^	3.03 ^a^	2.01 ^b^	1.24 ^c^
Cu	5.48 ^ab^	4.14 ^bc^	3.43 ^c^	7.04 ^a^	7.60 ^a^	6.62 ^a^	3.54 ^b^
Fe	94.2 ^a^	23.0 ^c^	22.7 ^c^	71.0 ^b^	94.1 ^a^	27.8 ^b^	24.1 ^b^
Hf	0.01 ^c^	0.02 ^b^	0.02 ^b^	0.03 ^a^	0.02 ^c^	0.03 ^a^	0.02 ^a^
Hg	0.03 ^b^	0.01 ^c^	0.01 ^c^	0.03 ^a^	0.04 ^a^	0.02 ^b^	0.01 ^b^
In	0.07 ^a^	<0.01 ^b^	<0.01 ^b^	0.06 ^a^	0.04 ^a^	<0.01 ^b^	<0.01 ^b^
Li	0.03 ^b^	0.01 ^c^	0.01 ^c^	0.10 ^a^	0.06 ^a^	0.02 ^b^	0.01 ^b^
Mn	1.42 ^b^	1.37 ^b^	1.04 ^b^	4.83 ^a^	1.86 ^a^	1.65 ^a^	1.12 ^b^
Mo	0.06 ^c^	0.20 ^ab^	0.12 ^b^	0.23 ^a^	0.11 ^c^	0.26 ^a^	0.16 ^b^
Ni	0.11 ^b^	0.13 ^b^	0.13 ^b^	0.32 ^a^	0.17 ^ab^	0.20 ^a^	0.14 ^b^
Pb	0.46 ^d^	3.37 ^b^	2.02 ^c^	9.36 ^a^	0.79 ^c^	4.59 ^a^	2.17 ^b^
Rb	0.31 ^c^	0.49 ^b^	0.28 ^c^	0.78 ^a^	0.50 ^b^	0.61 ^a^	0.30 ^c^
Re	0.06 ^b^	0.07 ^b^	0.07 ^b^	0.22 ^a^	0.06 ^b^	0.11 ^a^	0.05 ^b^
Sb	0.02 ^b^	<0.01 ^c^	0.01 ^c^	0.05 ^a^	0.04 ^a^	0.03 ^a^	0.01 ^b^
Se	<0.01 ^b^	<0.01 ^b^	<0.01 ^b^	0.02 ^a^	<0.01 ^a^	<0.01 ^a^	<0.01 ^a^
Si	7.12 ^a^	1.95 ^b^	2.16 ^b^	7.86 ^a^	9.71 ^a^	5.32 ^b^	2.10 ^c^
Sn	<0.01 ^c^	0.06 ^ab^	0.04 ^b^	0.09 ^a^	<0.01 ^c^	0.08 ^a^	0.04 ^b^
Sr	2.05 ^c^	2.74 ^b^	1.90 ^c^	5.78 ^a^	2.83 ^a^	3.16 ^a^	1.72 ^b^
Ta	0.16 ^b^	0.08 ^c^	0.02 ^c^	0.18 ^a^	0.15 ^a^	0.12 ^b^	0.05 ^c^
Te	0.11 ^c^	0.18 ^b^	0.14 ^bc^	0.26 ^a^	0.14 ^b^	0.28 ^a^	0.16 ^b^
Th	<0.01 ^c^	0.03 ^b^	0.03 ^b^	0.10 ^a^	0.05 ^b^	0.06 ^a^	0.03 ^c^
Ti	0.11 ^b^	0.61 ^b^	0.55 ^b^	1.39 ^a^	0.15 ^c^	0.94 ^a^	0.57 ^b^
Tl	<0.01 ^b^	<0.01 ^b^	<0.01 ^b^	0.67 ^a^	<0.01 ^a^	<0.01 ^a^	<0.01 ^a^
V	0.03 ^bc^	0.04 ^b^	0.01 ^c^	0.07 ^a^	0.06 ^a^	0.05 ^a^	0.02 ^b^
W	0.22 ^b^	0.13 ^c^	0.06 ^d^	0.31 ^a^	0.30 ^a^	0.21 ^b^	0.07 ^c^
Zn	1.05 ^bc^	1.28 ^b^	0.89 ^c^	5.04 ^a^	1.44 ^a^	1.47 ^a^	0.93 ^a^
Zr	0.03 ^b^	0.02 ^c^	0.01 ^c^	0.05 ^a^	0.05 ^a^	0.04 ^a^	0.02 ^a^
NE	1.53 ^c^	3.61 ^b^	3.48 ^b^	12.0 ^a^	1.85 ^b^	4.53 ^a^	3.72 ^a^
REEs	0.14 ^b^	0.10 ^bc^	0.05 ^c^	0.38 ^a^	0.15 ^a^	0.13 ^a^	0.06 ^b^

Identical superscripts denote no significant (*p* > 0.05) differences between elements content in whole plant biomass (within a row, separately for particular years of samples collection) according to a post-hoc Tukey’s HDS test.

**Table 4:** Metal extraction ratio (MER) values (%) for major and trace elements determined in *Paulownia* plants growing under particular experimental systems ("-“ means MER value below 0.001).

Element(s)	2018				2019		
	Control	FT	FT/BR	MS	Control	FT	FT/BR
Ca	0.332	0.021	0.164	0.038	0.355	0.017	0.122
K	0.423	0.044	0.041	0.074	0.479	0.036	0.029
Mg	0.107	0.004	0.003	0.007	0.155	0.005	0.003
Na	0.034	0.022	0.010	0.026	0.062	0.049	0.009
P	0.213	0.028	0.014	0.029	0.486	0.032	0.015
Al	0.001	-	-	0.001	0.002	-	-
As	-	0.002	0.001	-	0.002	0.002	0.009
B	0.661	0.185	0.202	0.031	0.403	0.134	0.131
Ba	0.004	0.002	0.002	-	0.005	0.002	0.002
Bi	0.004	0.011	0.014	0.005	0.005	0.019	0.013
Cd	0.001	0.005	0.003	-	0.001	0.006	0.003
Co	0.012	0.002	0.001	-	0.011	0.002	0.001
Cr	0.005	0.001	-	-	0.005	0.001	-
Cs	0.010	0.001	0.001	0.002	0.011	0.001	0.001
Cu	0.185	-	-	-	0.389	-	-
Fe	0.019	0.001	0.001	-	0.018	0.001	-
Hf	-	0.016	0.012	0.003	-	0.016	0.012
Hg	0.006	0.003	0.003	-	0.007	0.005	0.003
In	0.018	-	-	0.004	0.014	-	-
Li	0.001	-	-	0.002	0.002	-	-
Mn	0.005	0.001	0.001	0.006	0.005	0.001	0.001
Mo	0.004	0.013	0.026	0.013	0.004	0.016	0.021
Ni	0.010	0.002	0.001	-	0.012	0.002	0.001
Pb	0.007	0.025	0.018	0.001	0.012	0.028	0.018
Rb	0.025	0.010	0.008	0.035	0.056	0.011	0.008
Re	0.032	0.083	0.093	0.235	0.046	0.096	0.063
Si	0.007	0.002	0.001	0.006	0.007	0.003	0.001
Sn	-	0.004	0.004	0.001	0.000	0.004	0.003
Sr	0.230	0.009	0.007	0.041	0.320	0.009	0.006
Ta	0.097	0.094	0.011	0.046	0.126	0.093	0.045
Te	0.012	0.014	0.009	0.014	0.015	0.018	0.010
Th	0.012	0.001	0.002	0.003	0.016	0.003	0.002
W	0.033	0.038	0.017	-	0.051	0.045	0.023
Zn	0.025	0.024	0.015	-	0.038	0.023	0.016
Zr	0.002	0.001	0.001	-	0.002	0.001	0.001
NE	0.012	0.007	0.009	0.001	0.014	0.006	0.007
REEs	0.001	0.001	-	0.001	0.001	-	-

**Table 5:** Plant Effective Number (PEN) for major and trace elements determined in *Paulownia* plants growing under particular experimental systems ("-“ means PEN value ≥ 10^7^).

Element(s)	2018				2019		
	Control	FT	FT/BR	MS/BR	Control	FT	FT/BR
Ca	1	1	2	1	1	2	3
K	3	2	3	3	4	3	5
Mg	15	25	60	14	19	37	95
Na	76	33	184	30	74	43	305
P	6	64	115	52	7	71	190
Al	93	179	301	107	162	240	397
As	180,883	11,782	17,260	1038	41,915	11,700	25,006
B	397	985	1467	244	868	1294	2416
Ba	1067	503	721	395	1129	612	1173
Be	-	1155,108	1831,804	1175,157	-	1023,751	2986,858
Bi	67,095	51,587	31,304	22,804	59,872	28,783	52,709
Cd	454,868	81,371	178,572	5798	343,407	85,800	258,478
Co	29,346	35,975	82,298	32,583	37,125	46,510	126,502
Cr	21,346	46,651	132,368	17,746	25,890	51,509	159,535
Cs	312	558	945	492	304	762	1487
Cu	522	271	310	194	435	369	557
Fe	12	100	177	69	12	141	286
Hf	-	25,375	38,291	84,883	-	31,722	54,749
Hg	47,368	72,490	137,496	20,261	48,563	81,975	199,864
In	11,248	-	-	34,008	13,581	-	-
Li	147,995	87,681	148,266	20,714	116,128	96,560	211,251
Mn	874	742	1291	138	950	1044	2212
Mo	78,306	7309	7876	2938	67,619	7295	11,895
Ni	21,244	22,982	46,584	5283	20,741	27806	72,263
Pb	3252	351	591	607	2730	504	1051
Rb	4515	1325	1945	899	2988	1756	3403
Re	9345	5271	5818	1760	8067	4649	9661
Si	256	408	737	133	217	369	1310
Sn	-	5271	8413	3693		6188	13,475
Sr	427	214	296	87	361	255	494
Ta	3539	3856	22,079	3148	3329	5347	14,337
Te	8114	4526	11,069	1843	7374	4767	13,965
Th	14,195	38,199	35,536	8784	10,207	22,912	49,506
Ti	10,980	11,834	16,860	2524	13,815	13,000	25,164
Tl	-	-	-	1853	-	-	-
V	154,655	257,937	469,305	290,561	177,406	173,919	607,497
W	4152	3971	9218	2763	3596	4904	13,821
Zn	1279	434	709	220	1023	642	1146
Zr	62,235	64,720	75,715	32,245	55,774	60,544	118,032
NE	747	843	1154	698	791	1096	1844
REEs	13,095	17,124	26,265	6016	16,188	23,639	44,323

## Data Availability

Not applicable.

## Supplementary Materials

The following are available online at https://www.mdpi.com/article/10.3390/plants10102049/s1, Table S1: Concentration of major and trace element(s) [mg kg^−1^ DW] in particular fractions of used substrates, Table S2: Content of major and trace elements [mg kg^−1^ DW] in root, stem and leaves of *Paulownia* growing under particular experimental systems after the first year of experiment, Table S3: Content of major and trace elements (mg kg^−1^ DW) in root, stem and leaves of *Paulownia* growing under particular experimental systems after the second year of experiment, Table S4: Biochemical and physiological parameters of plants cultivated on soil (control) and waste materials (FT—flotation tailings, FT/BR—flotation tailings supplemented with biochar, MS/BR—mining sludge supplemented with biochar).
