# Supplementary material for: The Possibility of Using Paulownia elongata S. Y. Hu × Paulownia fortunei Hybrid for Phytoextraction of Toxic Elements from Post-Industrial Wastes with Biochar
Source: Plants (Basel). 2021 Sep 29;10(10):2049. doi: 10.3390/plants10102049 (PMC8541643; doi:10.3390/plants10102049)
Supplement: Supplementary file 1 [file plants-10-02049-s001.zip › plants-1365924-supplementary.pdf]

Table S1. Concentration of major and trace elements [mg kg^{-1} DW] in particular fractions of used substrates

Element(s)	Fraction	Control	FT	FT/BR	MS	MS/BR
Ca	F1	541^{a}	21100^{a}	20400^{a}	14300^{a}	13800^{a}
	F2	72.0^{b}	1670^{c}	1620^{c}	8070^{b}	7980^{b}
	F3	114^{b}	604^{d}	589^{d}	288^{d}	272^{d}
	F4	512^{a}	3490^{b}	3520^{b}	685^{c}	652^{c}
K	F1	9.96^{b}	593^{b}	591^{b}	2270^{a}	2160^{a}
	F2	10.2^{b}	171^{c}	176^{c}	88.8^{c}	51.0^{c}
	F3	0.47^{c}	88.2^{d}	86.0^{d}	75.4^{c}	29.3^{c}
	F4	258^{a}	6320^{a}	5990^{a}	726^{b}	708^{b}
Mg	F1	19.1^{b}	2620^{a}	2570^{a}	2710^{a}	2780^{a}
	F2	2.49^{c}	373^{c}	321^{c}	125^{c}	109^{c}
	F3	6.14^{c}	347^{c}	298^{c}	71.0^{c}	69.2^{c}
	F4	160^{a}	847^{b}	829^{b}	305^{b}	259^{b}
Na	F1	51.7^{a}	228^{a}	228^{a}	392^{a}	306^{a}
	F2	0.546^{c}	4.83^{d}	3.71^{d}	43.4^{c}	37.9^{d}
	F3	28.8^{b}	40.2^{c}	49.0^{c}	83.5^{b}	75.0^{c}
	F4	36.8^{b}	110^{b}	84.5^{b}	46.8^{c}	56.7^{b}
P	F1	182^{a}	101^{ab}	118^{a}	135^{a}	132^{a}
	F2	75.0^{b}	126^{a}	113^{a}	136^{a}	135^{a}
	F3	123^{ab}	79.4^{b}	74.5^{b}	43.0^{b}	36.9^{c}
	F4	57.7^{b}	43.7^{c}	40.2^{c}	125^{a}	101^{b}
Al	F1	423^{b}	1200^{b}	1490^{b}	5030^{a}	4990^{a}
	F2	164^{c}	679^{c}	624^{c}	560^{d}	502^{d}
	F3	210^{c}	844^{bc}	671^{c}	1130^{c}	1120^{c}
	F4	1420^{a}	15400^{a}	14900^{a}	2500^{b}	2520^{b}
As	F1	4.56^{a}	12.1^{a}	15.8^{a}	5640^{a}	5280^{a}
	F2	0.38^{c}	7.59^{b}	1.56^{c}	3210^{b}	3260^{b}
	F3	0.63^{bc}	4.09^{c}	5.20^{b}	690^{d}	634^{d}
	F4	0.89^{b}	7.31^{b}	2.92^{bc}	1960^{c}	1890^{c}
B	F1	0.38^{b}	0.90^{b}	0.39^{b}	98.3^{a}	88.0^{a}
	F2	0.38^{b}	0.39^{c}	0.39^{b}	4.94^{c}	1.22^{c}
	F3	0.47^{a}	0.49^{c}	0.49^{b}	1.06^{c}	1.54^{c}
	F4	0.37^{b}	2.70^{a}	1.73^{a}	12.9^{b}	19.2^{b}
Ba	F1	14.2^{c}	35.2^{c}	44.1^{b}	3770^{a}	3660^{a}
	F2	14.1^{c}	45.9^{bc}	44.2^{b}	191^{c}	143^{c}
	F3	22.2^{b}	74.2^{b}	69.0^{b}	50.4^{d}	42.2^{d}
	F4	41.5^{a}	409^{a}	386^{a}	735^{b}	704^{b}
Be	F1	0.386^{b}	0.391^{b}	0.392^{b}	0.368^{ab}	0.384^{a}
	F2	0.383^{b}	0.390^{b}	0.393^{b}	0.371^{ab}	0.383^{a}
	F3	0.478^{a}	0.494^{ab}	0.498^{a}	0.458^{a}	0.428^{a}
	F4	0.042^{c}	0.568^{a}	0.365^{b}	0.27^{b}	0.113^{b}
Bi	F1	0.55^{a}	0.39^{b}	0.39^{a}	1.67^{a}	1.22^{b}
	F2	0.47^{a}	0.39^{b}	0.39^{a}	1.44^{ab}	1.63^{a}
	F3	0.47^{a}	0.51^{a}	0.49^{a}	1.48^{ab}	1.61^{a}
	F4	0.18^{b}	0.18^{c}	0.14^{b}	1.03^{b}	0.921^{c}
Cd	F1	0.38^{b}	0.46^{ab}	0.39^{b}	1550^{a}	1520^{a}
	F2	0.38^{b}	0.39^{b}	0.39^{b}	124^{b}	106^{b}
	F3	0.47^{a}	0.49^{a}	0.49^{a}	42.6^{c}	31.0^{c}
	F4	0.08^{c}	0.20^{c}	0.08^{c}	14.7^{c}	13.0^{c}

Mean values (n=3); identical superscripts denote no significant ($p>0.05$) differences between content of particular elements in substrate fractions (in columns) according to a post-hoc Tukey's HDS test.

Table S1. Concentration of major and trace elements [mg kg^{-1} DW] in particular fractions of used substrates

Element(s)	Fraction	Control	FT	FT/BR	MS	MS/BR
Co	F1	0.38^{b}	4.43^{a}	2.92^{a}	82.0^{a}	74.1^{a}
	F2	0.38^{b}	0.39^{b}	2.07^{b}	1.69^{c}	1.18^{c}
	F3	0.47^{a}	3.25^{a}	2.60^{ab}	17.1^{b}	18.2^{b}
	F4	0.07^{c}	0.58^{b}	0.64^{c}	1.26^{c}	2.42^{c}
Cr	F1	0.38^{c}	4.85^{b}	3.18^{b}	194^{a}	210^{a}
	F2	0.38^{c}	0.39^{c}	0.142^{c}	56.9^{c}	50.8^{c}
	F3	1.28^{b}	1.34^{c}	3.26^{b}	225^{a}	211^{a}
	F4	2.22^{a}	14.9^{a}	14.8^{a}	143^{ab}	144^{b}
Cs	F1	24.0^{b}	650^{a}	616^{a}	458^{a}	386^{a}
	F2	48.9^{ab}	137^{b}	162^{b}	30.6^{c}	52.7^{bc}
	F3	55.9^{a}	98.0^{bc}	82.8^{c}	44.8^{bc}	32.6^{d}
	F4	28.2^{b}	48.2^{c}	39.8^{d}	52.5^{b}	56.9^{b}
Cu	F1	0.95^{a}	4440^{a}	4250^{a}	5840^{a}	5800^{a}
	F2	0.44^{b}	406^{b}	403^{b}	288^{c}	293^{c}
	F3	0.90^{a}	142^{c}	108^{c}	1420^{b}	1330^{b}
	F4	0.89^{a}	21.4^{d}	20.4^{d}	318^{c}	287^{c}
Fe	F1	48.9^{d}	1060^{b}	1040^{d}	3620^{b}	3440^{c}
	F2	269^{b}	922^{bc}	729^{b}	7550^{a}	7370^{b}
	F3	185^{c}	772^{c}	740^{c}	1980^{c}	1790^{d}
	F4	2020^{a}	4800^{a}	4780^{a}	14700^{a}	14700^{a}
Ga	F1	0.381^{b}	0.894^{b}	0.784^{b}	1.08^{b}	1.03^{b}
	F2	0.383^{b}	0.391^{c}	0.816^{b}	1.06^{b}	1.03^{b}
	F3	0.470^{ab}	0.992^{b}	0.982^{b}	1.50^{b}	1.30^{b}
	F4	0.686^{a}	3.26^{a}	2.22^{a}	7.49^{a}	6.96^{a}
Ge	F1	0.382^{c}	1.18^{a}	0.832^{b}	8.45^{a}	7.49^{a}
	F2	1.82^{a}	0.583^{b}	1.11^{a}	4.47^{b}	4.43^{b}
	F3	0.475^{bc}	0.492^{b}	0.495^{c}	3.21^{bc}	3.14^{bc}
	F4	0.833^{b}	1.244^{a}	0.951^{b}	2.11^{c}	1.76^{c}
Hf	F1	0.389^{a}	0.393^{ab}	0.390^{ab}	0.384^{b}	0.383^{ab}
	F2	0.386^{a}	0.394^{ab}	0.391^{ab}	0.386^{b}	0.384^{ab}
	F3	0.470^{a}	0.495^{a}	0.496^{a}	0.818^{a}	0.576^{a}
	F4	0.102^{b}	0.230^{b}	0.208^{b}	0.410^{b}	0.191^{b}
Hg	F1	0.402^{ab}	0.785^{a}	0.582^{a}	6.45^{c}	5.67^{c}
	F2	0.382^{ab}	0.390^{b}	0.391^{b}	0.382^{d}	0.380^{d}
	F3	0.563^{a}	0.574^{ab}	0.514^{ab}	15.4^{b}	15.0^{b}
	F4	0.191^{b}	0.151^{c}	0.149^{c}	48.7^{a}	46.2^{a}
In	F1	1.31^{a}	1.77^{a}	1.36^{a}	1.79^{a}	1.75^{a}
	F2	0.402^{c}	0.884^{c}	0.39^{d}	1.27^{c}	1.08^{c}
	F3	1.01^{b}	0.942^{b}	0.91^{b}	1.55^{b}	1.24^{b}
	F4	0.269^{c}	0.463^{d}	0.50^{c}	1.34^{bc}	1.19^{bc}
Li	F1	0.38^{b}	1.35^{b}	2.57^{b}	9.24^{a}	8.81^{a}
	F2	0.38^{b}	1.89^{c}	2.57^{b}	0.76^{b}	0.460^{b}
	F3	0.47^{b}	2.33^{b}	3.23^{b}	1.04^{b}	0.555^{b}
	F4	1.27^{a}	19.0^{a}	15.5^{a}	8.58^{a}	7.95^{a}
Mn	F1	37.4^{b}	926^{a}	371^{a}	607^{a}	592^{a}
	F2	20.7^{c}	9.60^{c}	244^{b}	53.7^{b}	43.8^{b}
	F3	11.7^{d}	9.73^{c}	25.2^{c}	34.2^{b}	36.8^{b}
	F4	46.4^{a}	24.3^{b}	233^{b}	36.0^{b}	24.5^{b}

Mean values (n=3); identical superscripts denote no significant ($p>0.05$) differences between content of particular elements in substrate fractions (in columns) according to a post-hoc Tukey's HDS test.

Table S1. Concentration of major and trace elements [mg kg^{-1} DW] in particular fractions of used substrates

Element(s)	Fraction	Control	FT	FT/BR	MS	MS/BR
Mo	F1	0.382^{b}	0.394^{c}	0.420^{bc}	0.382^{b}	0.384^{b}
	F2	0.434^{ab}	0.395^{c}	0.391^{c}	0.460^{b}	0.440^{b}
	F3	0.471^{ab}	3.49^{a}	0.549^{b}	0.477^{b}	0.483^{b}
	F4	0.609^{a}	2.33^{b}	2.39^{a}	18.1^{a}	16.9^{a}
Ni	F1	0.383^{b}	2.84^{b}	2.81^{b}	486^{a}	422^{a}
	F2	0.389^{b}	2.67^{c}	2.18^{b}	28.9^{b}	21.8^{b}
	F3	0.472^{b}	2.56^{a}	2.21^{b}	31.1^{b}	28.4^{b}
	F4	0.988^{a}	5.99^{a}	5.15^{a}	26.2^{b}	23.6^{b}
Pb	F1	3.24^{b}	20.1^{a}	17.8^{a}	1280^{a}	1250^{a}
	F2	5.73^{a}	16.8^{b}	16.4^{a}	270^{b}	271^{b}
	F3	4.18^{ab}	12.9^{b}	12.7^{b}	26.5^{d}	26.3^{d}
	F4	3.96^{ab}	6.97^{c}	6.53^{c}	89.0^{c}	98.6^{c}
Rb	F1	0.383^{b}	2.86^{b}	3.12^{b}	0.389^{b}	0.382^{b}
	F2	0.388^{b}	2.79^{b}	3.02^{b}	0.388^{b}	0.384^{b}
	F3	0.479^{b}	2.49^{b}	3.28^{b}	0.485^{b}	0.479^{b}
	F4	1.98^{a}	34.3^{a}	28.4^{a}	16.9^{a}	16.9^{a}
Re	F1	0.415^{ab}	0.522^{a}	0.496^{b}	0.815^{a}	0.580^{a}
	F2	0.386^{ab}	0.463^{b}	0.387^{b}	0.381^{b}	0.384^{b}
	F3	0.471^{a}	0.494^{ab}	0.491^{a}	0.632^{ab}	0.479^{ab}
	F4	0.210^{b}	0.266^{c}	0.189^{c}	0.196^{c}	0.192^{c}
Sb	F1	2.03^{a}	2.16^{a}	0.963^{b}	32.8^{b}	29.4^{b}
	F2	0.682^{b}	0.655^{bc}	0.472^{c}	19.2^{c}	18.5^{c}
	F3	1.01^{a}	1.40^{b}	1.49^{a}	29.9^{bc}	26.4^{bc}
	F4	0.644^{b}	0.635^{c}	0.951^{b}	147^{a}	133^{a}
Se	F1	9.38^{a}	11.0^{a}	9.24^{a}	50.0^{a}	46.9^{a}
	F2	0.754^{b}	1.64^{b}	0.613^{b}	3.54^{b}	2.78^{b}
	F3	9.40^{a}	11.9^{a}	9.91^{a}	52.4^{a}	51.7^{a}
	F4	0.72^{b}	1.04^{b}	0.512^{b}	1.06^{b}	0.88^{b}
Si	F1	21.4^{c}	242^{ab}	213^{ab}	290^{a}	215^{a}
	F2	11.7^{c}	85.1^{c}	83.3^{c}	64.2^{b}	52.9^{b}
	F3	63.7^{b}	163^{b}	136^{b}	81.0^{b}	74.0^{b}
	F4	251^{a}	329^{a}	311^{a}	415^{a}	381^{b}
Sn	F1	0.532^{ab}	15.0^{a}	3.90^{b}	80.1^{a}	77.5^{a}
	F2	0.664^{ab}	3.48^{c}	4.62^{b}	1.71^{c}	1.47^{c}
	F3	0.576^{a}	3.49^{c}	4.90^{b}	88.2^{a}	83.1^{a}
	F4	0.312^{b}	9.71^{b}	9.12^{a}	9.17^{b}	8.58^{b}
Sr	F1	1.27^{b}	322^{a}	303^{a}	160^{a}	128^{a}
	F2	0.384^{c}	7.62^{c}	8.02^{c}	13.3^{b}	10.4^{b}
	F3	0.473^{c}	8.88^{c}	8.13^{c}	7.94^{b}	8.69^{b}
	F4	2.61^{a}	22.4^{b}	20.6^{b}	11.4^{b}	4.64^{b}
Ta	F1	0.362^{a}	0.442^{ab}	0.398^{a}	0.486^{b}	1.08^{a}
	F2	0.385^{a}	0.395^{ab}	0.394^{a}	0.553^{b}	1.08^{a}
	F3	0.473^{a}	0.493^{a}	0.495^{a}	0.517^{b}	1.28^{a}
	F4	0.086^{b}	0.263^{b}	0.202^{b}	2.499^{a}	0.386^{b}

Mean values (n=3); identical superscripts denote no significant ($p>0.05$) differences between content of particular elements in substrate fractions (in columns) according to a post-hoc Tukey's HDS test.

Table S1. Concentration of major and trace elements [mg kg^{-1} DW] in particular fractions of used substrates

Element(s)	Fraction	Control	FT	FT/BR	MS	MS/BR
Te	F1	2.12^{a}	4.06^{a}	1.96^{ab}	12.8^{a}	5.17^{b}
	F2	1.09^{ab}	1.94^{b}	1.21^{b}	2.20^{c}	3.80^{c}
	F3	1.36^{ab}	2.06^{b}	2.53^{a}	1.13^{c}	9.78^{a}
	F4	0.583^{b}	1.09^{b}	1.01^{b}	5.27^{b}	1.13^{c}
Th	F1	0.382^{b}	3.82^{ab}	3.39^{ab}	3.74^{b}	8.08^{b}
	F2	0.467^{b}	0.82^{b}	0.98^{b}	5.92^{b}	6.12^{bc}
	F3	0.545^{b}	1.57^{b}	1.34^{b}	3.55^{b}	3.68^{c}
	F4	2.09^{a}	6.52^{a}	6.48^{a}	17.8^{a}	14.7^{a}
Ti	F1	0.386^{c}	0.398^{c}	0.597^{b}	0.416^{b}	0.333^{b}
	F2	0.382^{c}	0.395^{c}	0.591^{b}	0.492^{b}	0.412^{b}
	F3	16.1^{b}	19.9^{b}	0.722^{b}	2.98^{b}	2.73^{b}
	F4	222^{a}	329^{a}	314^{a}	330^{a}	319^{a}
Tl	F1	0.522^{b}	1.24^{a}	0.975^{b}	42.6^{b}	42.7^{b}
	F2	1.44^{a}	1.35^{a}	1.41^{a}	91.5^{a}	89.6^{a}
	F3	0.533^{b}	0.522^{b}	0.492^{c}	40.5^{b}	36.7^{b}
	F4	0.774^{b}	1.05^{a}	1.12^{ab}	16.4^{c}	12.9^{c}
V	F1	1.32^{b}	1.14^{c}	1.09^{c}	35.7^{b}	37.4^{b}
	F2	0.942^{c}	2.06^{bc}	1.43^{bc}	20.2^{bc}	19.2^{c}
	F3	1.94^{b}	5.49^{b}	5.40^{b}	8.91^{c}	6.55^{d}
	F4	3.17^{a}	16.8^{a}	15.8^{a}	57.8^{a}	59.3^{a}
W	F1	0.755^{b}	0.519^{c}	0.565^{b}	1070^{a}	1060^{a}
	F2	0.802^{ab}	1.22^{a}	0.643^{ab}	62.7^{b}	77.5^{b}
	F3	0.880^{a}	0.973^{b}	1.38^{a}	46.7^{b}	44.5^{b}
	F4	0.567^{c}	0.872^{bc}	0.562^{b}	51.0^{b}	40.8^{b}
Zn	F1	5.73^{a}	26.4^{a}	25.2^{a}	6600^{a}	6540^{a}
	F2	1.22^{b}	4.09^{c}	4.35^{b}	2880^{b}	2690^{b}
	F3	1.60^{b}	3.81^{c}	4.52^{b}	1970^{bc}	2020^{bc}
	F4	5.62^{a}	19.4^{b}	18.8^{a}	1060^{c}	1050^{c}
Zr	F1	0.388^{b}	0.391^{b}	0.394^{b}	0.573^{b}	3.19^{b}
	F2	0.368^{b}	0.396^{b}	0.396^{b}	1.61^{b}	3.19^{b}
	F3	0.427^{b}	0.492^{b}	0.492^{b}	32.0^{a}	24.3^{a}
	F4	2.90^{a}	7.38^{a}	5.81^{a}	31.5^{a}	32.9^{a}
NEs	F1	6.81^{d}	15.5^{c}	7.16^{d}	37.2^{b}	72.8^{a}
	F2	7.53^{d}	15.2^{c}	8.86^{d}	91.0^{b}	148^{a}
	F3	6.36^{d}	20.0^{c}	8.05^{d}	29.3^{b}	69.0^{a}
	F4	29.1^{d}	64.1^{c}	52.1^{cd}	650^{a}	245^{b}
REEs	F1	6.02^{c}	43.2^{a}	36.4^{a}	22.8^{b}	23.4^{a}
	F2	6.02^{c}	7.69^{c}	7.77^{c}	10.9^{c}	6.70^{c}
	F3	7.55^{b}	11.6^{c}	9.27^{c}	12.1^{c}	11.9^{b}
	F4	12.9^{a}	20.4^{b}	18.8^{b}	30.6^{a}	23.8^{a}

Mean values (n=3); identical superscripts denote no significant ($p>0.05$) differences between content of particular elements in substrate fractions (in columns) according to a post-hoc Tukey's HDS test.

Table S2. Content of major and trace elements [mg kg^{-1} DW] in root, stem and leaves of *Paulownia* growing under particular experimental systems after the first year of experiment

Element	Control			FT			FT/BR			MS/BR		
	Leaf	Stem	Root	Leaf	Stem	Root	Leaf	Stem	Root	Leaf	Stem	Root
Ca	16600^{a}	12600^{a}	5650^{b}	32700^{a}	9820^{b}	8900^{b}	23500^{a}	9350^{b}	7690^{b}	47800^{a}	15600^{b}	12400^{b}
K	4367^{a}	4700^{a}	3930^{a}	17700^{a}	7250^{b}	6010^{b}	15400^{a}	5830^{b}	5020^{b}	11900^{a}	7780^{a}	10700^{a}
Mg	433^{b}	1630^{a}	1770^{a}	737^{c}	1360^{b}	2430^{a}	564^{b}	390^{b}	1110^{a}	722^{c}	3030^{b}	4930^{a}
Na	92.6^{b}	317^{b}	1420^{a}	186^{c}	1430^{b}	2440^{a}	179^{b}	129^{b}	2870^{a}	598^{b}	1170^{b}	2980^{a}
P	4420^{a}	1090^{b}	551^{c}	526^{a}	307^{b}	142^{b}	227^{a}	267^{a}	312^{a}	665^{a}	359^{a}	389^{a}
Al	175^{b}	159^{b}	559^{a}	203^{b}	94.3^{c}	1130^{a}	123^{b}	65.4^{b}	702^{a}	362^{b}	132^{b}	3590^{a}
As	0.104^{b}	0.067^{b}	3.07^{a}	2.76^{ab}	1.75^{b}	5.54^{a}	1.66^{a}	1.63^{a}	2.13^{a}	45.2^{b}	5.77^{b}	68.4^{a}
B	41.0^{a}	37.0^{a}	2.76^{b}	50.3^{a}	3.65^{b}	4.63^{b}	37.0^{a}	1.72^{b}	4.69^{c}	206^{a}	10.6^{b}	15.5^{b}
Ba	14.7^{a}	14.3^{a}	5.66^{b}	65.8^{a}	39.8^{b}	22.6^{c}	55.1^{a}	23.7^{b}	20.1^{b}	107^{a}	27.0^{b}	25.1^{b}
Be	<0.01^{b}	<0.01^{b}	0.068^{a}	0.014^{c}	0.032^{b}	0.055^{a}	0.010^{b}	0.021^{b}	0.052^{a}	0.021^{b}	0.024^{b}	0.085^{a}
Bi	0.240^{a}	0.221^{a}	<0.01^{b}	1.02^{a}	<0.01^{b}	<0.01^{b}	0.952^{a}	0.862^{a}	<0.01^{b}	1.53^{a}	0.789^{b}	<0.01^{c}
Cd	0.046^{b}	0.022^{b}	0.094^{a}	0.412^{a}	0.241^{b}	0.135^{b}	0.242^{a}	0.076^{b}	0.122^{b}	2.30^{b}	6.82^{b}	64.0^{a}
Co	0.638^{a}	0.416^{ab}	0.240^{b}	0.845^{b}	0.632^{b}	3.29^{a}	0.36^{b}	0.33^{b}	1.97^{a}	0.733^{b}	0.890^{b}	7.10^{a}
Cr	1.02^{ab}	0.429^{b}	1.36^{a}	0.697^{b}	0.442^{b}	2.17^{a}	0.257^{b}	0.172^{b}	2.56^{a}	1.86^{b}	1.12^{c}	5.63^{a}
Cs	65.2^{a}	34.0^{b}	34.7^{b}	77.7^{a}	17.6^{b}	67.9^{a}	46.8^{b}	13.3^{c}	62.8^{a}	64.7^{b}	42.8^{c}	164^{a}
Cu	13.4^{b}	45.8^{b}	201^{a}	65.3^{c}	131^{b}	235^{a}	55.3^{c}	128^{b}	184^{a}	108^{c}	164^{b}	365^{a}
Fe	2460^{a}	64.6^{c}	1410^{b}	427^{b}	103^{c}	1420^{a}	279^{b}	42.6^{c}	1500^{a}	636^{b}	129^{c}	4240^{a}
Hf	<0.01^{b}	<0.01^{b}	0.411^{a}	1.33^{a}	0.764^{b}	0.692^{b}	0.896^{a}	0.587^{a}	0.635^{a}	0.315^{b}	0.308^{b}	1.62^{a}
Hg	0.325^{b}	0.328^{b}	0.752^{a}	0.301^{b}	0.432^{ab}	0.531^{a}	0.244^{ab}	0.169^{b}	0.388^{a}	1.60^{a}	1.01^{b}	1.18^{b}
In	2.74^{a}	<0.01^{b}	<0.01^{b}	<0.01	<0.01	<0.01	<0.01	<0.01	<0.01	1.39^{b}	0.165^{c}	2.86^{a}
Li	0.178^{b}	0.031^{b}	1.04^{a}	0.493^{a}	0.113^{b}	0.526^{a}	0.342^{a}	0.041^{b}	0.418^{a}	2.29^{b}	0.263^{c}	5.53^{a}
Mn	25.3^{a}	10.1^{b}	29.9^{a}	28.9^{c}	42.7^{b}	73.5^{a}	22.8^{b}	21.2^{b}	57.4^{a}	240^{a}	144^{b}	162^{b}
Mo	0.385^{b}	<0.01^{c}	2.26^{a}	4.62^{b}	2.65^{b}	11.3^{a}	5.66^{a}	1.55^{b}	5.44^{a}	13.0^{a}	5.00^{b}	7.18^{b}
Ni	1.02^{b}	0.436^{c}	3.64^{a}	1.43^{b}	0.882^{b}	8.70^{a}	0.325^{b}	0.894^{b}	8.78^{a}	4.86^{b}	5.15^{b}	17.1^{a}
Pb	3.75^{b}	5.76^{b}	13.4^{a}	69.6^{b}	81.6^{b}	188^{a}	43.5^{b}	52.6^{b}	109^{a}	40.3^{b}	46.8^{b}	574^{a}

Rb	2.72[b]	4.13[b]	8.76[a]	21.0[a]	19.1[a]	19.0[a]	15.9[a]	13.3[a]	7.66[b]	30.2[a]	28.6[a]	29.0[a]
Re	1.94[a]	1.37[a]	<0.01[b]	8.65[a]	1.43[b]	<0.01[b]	8.69[a]	1.07[b]	<0.01[b]	26.9[a]	3.15[b]	<0.01[c]
Sb	<0.01[b]	<0.01[b]	1.00[a]	<0.01	<0.01	<0.01	<0.01[b]	<0.01[b]	0.71[a]	<0.01[b]	<0.01[b]	3.53[a]
Se	<0.01	<0.01	<0.01	<0.01	<0.01	<0.01	<0.01	<0.01	<0.01	<0.01[b]	<0.01[b]	1.17[a]
Si	121[b]	19.6[c]	169[a]	74.5[ab]	55.7[b]	86.5[a]	59.7[b]	17.3[c]	120[a]	269[b]	129[c]	346[a]
Sn	<0.01	<0.01	<0.01	5.88[a]	4.20[a]	0.124[b]	4.22[a]	2.53[ab]	0.096[b]	10.4[a]	3.92[b]	<0.01[c]
Sr	46.7[a]	25.8[b]	28.8[b]	176[a]	72.1[b]	81.2[b]	126[a]	65.7[b]	50.4[b]	422[a]	189[b]	124[c]
Ta	5.32[a]	3.42[a]	<0.01[b]	7.85[a]	5.93[a]	0.245[b]	0.422[b]	2.15[a]	0.885[b]	9.11[a]	7.69[b]	5.53[c]
Te	2.97[a]	0.842[b]	1.44[b]	6.93[a]	4.81[a]	8.39[a]	3.16[b]	1.97[b]	7.77[a]	14.1[a]	14.6[a]	6.86[b]
Th	2.04[a]	0.139[b]	<0.01[b]	1.05[a]	0.341[b]	1.63[a]	1.24[a]	0.358[b]	1.34[a]	4.45[a]	1.57[b]	3.78[ab]
Ti	1.97[a]	0.847[b]	2.51[a]	3.26[b]	1.23[b]	42.5[a]	2.85[b]	0.518[b]	38.5[a]	17.2[b]	3.75[b]	80.1[a]
Tl	<0.01	<0.01	<0.01	<0.01	<0.01	<0.01	<0.01	<0.01	<0.01	4.54[c]	24.0[b]	35.6[a]
V	<0.01[b]	0.190[b]	1.06[a]	0.082[b]	0.124[b]	2.51[a]	0.023[b]	0.098[b]	0.993[a]	0.172[b]	<0.01[b]	4.51[a]
W	3.70[a]	3.75[a]	3.92[a]	6.72[a]	6.66[a]	3.98[a]	2.12[b]	4.04[a]	1.86[b]	9.64[b]	9.50[b]	13.1[a]
Zn	15.2[ab]	8.98[b]	25.4[a]	64.3[a]	58.1[ab]	42.9[b]	36.4[a]	43.7[a]	32.5[a]	95.3[c]	145[b]	246[a]
Zr	0.412[a]	0.085[b]	0.674[a]	0.685[a]	0.136[b]	0.945[a]	0.582[a]	0.168[b]	0.536[a]	1.27[b]	0.370[c]	2.24[a]
NE	25.7[ab]	15.7[b]	32.4[a]	46.3[b]	16.7[c]	235[a]	37.7[b]	11.5[c]	231[a]	56.7[b]	19.1[b]	742[a]
REEs	1.52[b]	0.842[b]	3.95[a]	2.89[b]	0.213[c]	5.95[a]	1.95[a]	0.212[b]	2.85[a]	6.00[b]	2.79[c]	20.8[a]

Mean values (n=3); identical superscripts denote no significant (p>0.05) differences between content of particular elements in substrate fractions (in columns) according to a post-hoc Tukey's HDS test.

Table S3. Content of major and trace elements [mg kg^{-1} DW] in root, stem and leaves of *Paulownia* growing under particular experimental systems after the second year of experiment

Element	Control			FT			FT/BR		
	Leaf	Stem	Root	Leaf	Stem	Root	Leaf	Stem	Root
Ca	23200[a]	15300[b]	7160[c]	40500[a]	12700[b]	11500[b]	25500[a]	9850[b]	8050[b]
K	5670[a]	6100[a]	5870[a]	20100[a]	9800[b]	7630[b]	16400[a]	6020[b]	5130[b]
Mg	681[c]	1910[b]	3040[a]	917[b]	1510[b]	2760[a]	701[b]	427[c]	1250[a]
Na	205[b]	446[b]	1920[a]	293[c]	1800[b]	3660[a]	213[b]	140[b]	3060[a]
P	5560[a]	1340[b]	967[b]	822[a]	445[b]	404[b]	287[a]	280[a]	326[a]
Al	262[b]	34.3[c]	726[a]	247[b]	128[b]	1470[a]	190[b]	80.6[b]	887[a]
As	0.117[c]	1.03[b]	4.18[a]	3.91[b]	3.79[b]	7.01[a]	2.25[a]	2.05[a]	2.98[a]
B	49.5[a]	5.92[b]	4.98[b]	62.4[a]	7.21[b]	6.51[b]	41.1[a]	3.40[b]	5.84[b]
Ba	25.6[a]	17.0[b]	13.2[c]	104[a]	43.1[b]	31.1[b]	62.7[a]	29.0[b]	22.9[b]
Be	<0.01[b]	<0.01[b]	0.108[a]	0.022[c]	0.066[b]	0.097[a]	0.012[b]	0.024[b]	0.067[a]
Bi	0.416[a]	0.387[a]	<0.01[b]	1.31[b]	1.82[a]	<0.01[c]	1.00[a]	1.04[a]	<0.01[b]
Cd	0.081[b]	0.059[b]	0.139[a]	0.763[a]	0.287[b]	0.326[b]	0.318[a]	0.098[a]	0.141[a]
Co	0.710[a]	0.585[a]	0.892[a]	1.16[b]	0.777[b]	5.15[a]	0.436[b]	0.414[b]	2.44[a]
Cr	1.13[b]	0.727[b]	2.89[a]	1.17[b]	0.579[c]	3.77[a]	0.468[b]	0.206[b]	2.90[a]
Cs	91.4[a]	66.5[ab]	54.3[b]	93.9[a]	24.3[b]	104[a]	55.6[a]	16.7[b]	67.6[a]
Cu	27.4[c]	83.0[b]	254[a]	84.4[c]	160[b]	400[a]	61.1[c]	132[b]	193[a]
Fe	3880[a]	101[c]	2020[b]	508[b]	131[c]	1890[a]	320[b]	56.4[c]	1650[a]
Hf	<0.01[b]	<0.01[b]	0.950[a]	1.68[a]	1.16[b]	1.08[b]	1.23[a]	0.734[b]	0.796[ab]
Hg	0.432[b]	0.558[b]	1.24[a]	0.472[b]	0.627[ab]	1.06[a]	0.314[ab]	0.224[b]	0.475[a]
In	3.53[a]	<0.01[b]	<0.01[b]	<0.01	<0.01	<0.01	<0.01	<0.01	<0.01
Li	0.369[b]	0.045[b]	2.04[a]	0.751[a]	0.182[b]	1.03[a]	0.457[a]	0.052[b]	0.625[a]
Mn	35.2[b]	15.4[c]	51.9[a]	39.3[b]	47.0[b]	89.4[a]	24.9[b]	23.7[b]	65.6[a]
Mo	0.701[b]	<0.01[c]	4.17[a]	8.80[b]	3.55[c]	14.7[a]	7.42[a]	1.62[b]	8.96[a]
Ni	1.58[b]	0.738[b]	5.59[a]	1.99[b]	1.25[b]	14.1[a]	0.468[b]	1.02[b]	9.85[a]
Pb	7.31[c]	10.3[b]	23.9[a]	84.8[b]	94.1[b]	274[a]	46.3[b]	56.0[b]	124[a]

Rb	7.37^{b}	8.72^{b}	13.1^{a}	25.4^{a}	25.9^{a}	25.0^{a}	16.9^{a}	14.7^{ab}	11.4^{b}
Re	3.28^{a}	2.68^{b}	<0.01^{c}	17.2^{a}	2.18^{b}	<0.01^{b}	10.0^{a}	1.13^{b}	<0.01^{b}
Sb	<0.01^{b}	<0.01^{b}	1.60^{a}	<0.01^{b}	<0.01^{b}	2.44^{a}	<0.01^{b}	<0.01^{b}	0.99^{a}
Se	<0.01	<0.01	<0.01	<0.01	<0.01	<0.01	<0.01	<0.01	<0.01
Si	178^{b}	43.1^{c}	289^{a}	181^{b}	63.4^{c}	304^{a}	62.4^{b}	19.7^{c}	127^{a}
Sn	<0.01	<0.01	<0.01	7.95^{a}	6.61^{a}	0.167^{b}	4.33^{a}	3.65^{a}	0.114^{b}
Sr	71.9^{a}	61.1^{a}	56.8^{a}	246^{a}	107^{b}	93.2^{b}	137^{a}	80.5^{b}	53.5^{c}
Ta	8.07^{a}	6.37^{b}	<0.01^{c}	8.80^{a}	8.05^{a}	1.98^{b}	5.52^{a}	1.98^{b}	1.03^{b}
Te	4.59^{a}	1.93^{c}	2.85^{b}	10.8^{ab}	8.10^{b}	13.8^{a}	4.92^{b}	2.78^{b}	9.41^{a}
Th	4.27^{a}	0.44^{b}	<0.01^{b}	3.38^{a}	0.552^{b}	3.04^{a}	1.76^{a}	0.412^{b}	1.54^{a}
Ti	2.46^{ab}	1.02^{b}	4.49^{a}	5.11^{b}	1.82^{b}	68.5^{a}	3.62^{b}	0.653^{b}	40.6^{a}
Tl	<0.01	<0.01	<0.01	<0.01	<0.01	<0.01	<0.01	<0.01	<0.01
V	<0.01^{b}	0.261^{b}	2.36^{a}	0.126^{b}	0.392^{b}	3.67^{a}	0.042^{b}	0.135^{b}	1.12^{a}
W	7.31^{a}	6.06^{a}	6.30^{a}	9.26^{a}	9.11^{a}	8.14^{a}	3.09^{b}	4.69^{a}	2.54^{b}
Zn	30.7^{a}	16.3^{b}	37.1^{a}	72.1^{a}	68.3^{a}	53.0^{a}	42.5^{a}	51.3^{a}	36.2^{a}
Zr	0.597^{a}	0.265^{a}	1.46^{a}	1.16^{b}	0.328^{c}	2.34^{a}	0.670^{b}	0.241^{c}	0.986^{a}
NE	32.0^{b}	28.8^{b}	47.8^{a}	60.3^{b}	21.9^{b}	311^{a}	43.6^{b}	14.7^{b}	254^{a}
REEs	1.80^{b}	1.17^{b}	4.51^{a}	3.46^{b}	0.351^{c}	8.33^{a}	2.18^{b}	0.246^{c}	3.98^{a}

Mean values (n=3); identical superscripts denote no significant ($p>0.05$) differences between content of particular elements in substrate fractions (in columns) according to a post-hoc Tukey's HDS test.

Table S4. Biochemical and physiological parameters of plants cultivated on soil (control) and waste materials (FT – flotation tailings, FT/BR – flotation tailings supplemented with biochar, MS/BR – mining sludge supplemented with biochar)

Parameter	Unit	Control	FT	FT/BR	MS/BR	*p*
Chlorophyll a	mg g^{-1} FW	4.03±0.93	4.53±0.64	3.56±0.04	4.53±0.41	0.2254
Chlorophyll b	mg g^{-1} FW	1.72^{bc}±0.25	1.84^{b} ±0.08	1.47^{c}±0.04	2.06^{a}±0.08	0.0055
Carotenoids	mg g^{-1} FW	0.69^{c}±0.16	0.97^{ab}±0.15	0.89^{bc}±0.03	1.24^{a}±0.19	0.0123
a/b	-	2.33±0.20	2.45±0.25	2.42±0.09	2.21±0.29	0.5526
(a+b)/caro	-	8.37^{a}±0.21	6.60^{b}±0.30	5.70^{bc}±0.22	5.45^{c}±1.14	0.0015
TPC (l)	mg GA_{eq} g^{-1} FW	1.112^{a}±0.29	0.639^{c}±0.11	0.896^{ab}±0.05	0.788^{bc}±0.02	0.0080
RSC (l)	%	73.12^{a}±11.89	50.98^{b}±6.68	74.91^{a}±7.78	60.31^{b}±4.32	0.0040
2,5-DHBA (l)	µg g^{-1} FW	4.543^{a}±0.164	1.106^{b}±0.037	bDL	bDL	0.0000
Caffeic acid (l)	µg g^{-1} FW	0.196^{a}±0.017	0.0262^{b}±0.001	bDL	0.014^{b}±0.102	0.0000
p-Coumaric acid (l)	µg g^{-1} FW	0.801^{a}±0.014	0.013^{c}±0.001	0.034^{b}±0.001	bDL	0.0000
Chlorogenic acid (l)	µg g^{-1} FW	2.981^{a}±0.603	0.315^{b}±0.011	0.314^{b}±0.017	0.632^{b}±0.476	0.0001
Ferulic acid (l)	µg g^{-1} FW	6.277^{a}±0.036	0.074^{b}±0.003	0.117^{b}±0.004	bDL	0.0000
Sinapic acid (l)	µg g^{-1} FW	0.761^{a}±0.106	0.231^{b}±0.008	bDL	0.054^{c}±0.041	0.0000
Gallic acid (l)	µg g^{-1} FW	3.475±0.138	bDL	bDL	bDL	-
Protocatechuic acid (l)	µg g^{-1} FW	4.438±0.324	bDL	bDL	bDL	-
4-HBA (l)	µg g^{-1} FW	3.509±0.114	bDL	bDL	bDL	-
Vanillic acid (l)	µg g^{-1} FW	1.267^{b}±0.127	1.514^{a}±0.051	0.148^{c}±0.005	bDL	0.0000
Siringic acid (l)	µg g^{-1} FW	1.026^{a}±0.038	0.054^{b}±0.002	bDL	bDL	0.0000

t-Cinnamic acid (l)	µg g^{-1} FW	0.011^{b}±0.000	bDL	0.074^{a}±0.002	bDL	-
Salicylic acid (l)	ng g^{-1} FW	94.60^{b}± 5.81	55.49^{c}±3.98	45.68^{c}±2.44	129.46^{a}±16.98	0.0000
Rutin (l)	µg g^{-1} FW	5.463^{a}±0.448	0.214^{b}±0.007	0.329^{b}±0.002	bDL	0.0000
Quercetin (l)	µg g^{-1} FW	0.169^{a}±0.002	bDL	0.072^{b}±0.002	bDL	0.0000
Catechin (l)	µg g^{-1} FW	0.938±0.015	bDL	bDL	bDL	-
C6-C1 (l)	µg g^{-1} FW	18.274^{a}±0.046	2.677^{b}±0.009	0.302^{c}±0.009	0.130^{d}±0.017	0.0000
C6-C3 (l)	µg g^{-1} FW	5.378^{a}±0.804	0.661^{b}±0.022	0.583^{b}±0.018	0.700^{b}±0.537	0.0000
C6-C3-C6 (l)	µg g^{-1} FW	6.571^{a}±0.455	0.214^{b}±0.007	0.441^{b}±0.015	bDL	0.0000
2,5-DHBA (r)	µg g^{-1} FW	0.100^{b}±0.005	0.940^{a}±0.029	bDL	bDL	0.0000
Caffeic acid (r)	µg g^{-1} FW	0.061^{b}±0.003	0.724^{a}±0.309	0.051^{b}±0.062	bDL	0.0000
p-Coumaric acid (r)	µg g^{-1} FW	0.124^{a}±0.107	0.136^{a}±0.123	0.071^{b}±0.019	0.052^{b}±0.035	0.0000
Chlorogenic acid (r)	µg g^{-1} FW	0.773^{a}±0.041	0.121^{c}±0.006	0.156^{c}±0.032	0.351^{b}±0.031	0.0000
Ferulic acid (r)	µg g^{-1} FW	0.424^{a}±0.022	0.346^{b}±0.015	0.259^{c}±0.040	0.046^{d}±0.046	0.0000
Sinapic acid (r)	µg g^{-1} FW	0.359^{b}±0.019	0.704^{a}±0.094	0.205^{c}±0.039	0.079^{c}±0.002	0.0000
Gallic acid (r)	µg g^{-1} FW	1.744^{a}±0.092	1.028^{b}±0.070	0.367^{c}±0.017	0.251^{c}±0.048	0.0000
Protocatechuic acid (r)	µg g^{-1} FW	0.942^{a}±0.050	0.353^{b}±0.024	0.198^{c}±0.055	0.125^{c}±0.021	0.0000
4-HBA (r)	µg g^{-1} FW	0.787^{b}±0.041	1.263^{a}±0.208	0.472^{c}±0.075	0.346^{c}±0.021	0.0000
Vanillic acid (r)	µg g^{-1} FW	1.853^{a}±0.097	0.164^{b}±0.016	0.149^{b}±0.017	0.198^{b}±0.016	0.0000
Syringic acid (r)	µg g^{-1} FW	0.166^{a}±0.009	0.055^{b}±0.004	0.029^{c}±0.002	0.027^{c}±0.005	0.0000
t-Cinnamic acid (r)	µg g^{-1} FW	0.027^{a}±0.001	0.022^{b}±0.001	bDL	0.010^{c}±0.000	0.0000
Rutin (r)	µg g^{-1} FW	1.009^{b}±0.053	1.500^{a}±0.145	0.770^{c}±0.048	0.663^{c}±0.036	0.0000

Quercetin (r)	µg g^{-1} FW	1.952^{a}±0.103	0.579^{b}±0.033	0.288^{c}±0.021	0.054^{d}±0.010	0.0000
Catechin (r)	µg g^{-1} FW	0.474^{a}±0.025	0.587^{a}±0.091	0.209^{b}±0.048	0.028^{c}±0.005	0.0000
C6-C1 (r)	µg g^{-1} FW	5.927^{a}±0.313	4.132^{b}±0.437	1.558^{c}±0.068	1.032^{c}±0.015	0.0000
C6-C3 (r)	µg g^{-1} FW	1.769^{b}±0.093	2.054^{a}±0.121	0.708^{c}±0.033	0.549^{c}±0.080	0.0000
C6-C3-C6 (r)	µg g^{-1} FW	3.435^{a}±0.181	2.667^{b}±0.022	1.267^{c}±0.020	0.746^{d}±0.021	0.0000
Oxalic acid (r)	µg g^{-1} FW	0.042^{ab}±0.007	0.029^{bc}±0.008	0.019^{c}±0.004	0.047^{a}±0.009	0.0419
Quinic acid (r)	µg g^{-1} FW	0.688^{ab}±0.261	0.389^{b}±0.189	1.354^{a}±0.301	0.333^{b}±0.292	0.0382
Malic acid (r)	µg g^{-1} FW	1.197^{b}±0.477	1.525^{b}±0.108	6.664^{a}±1.069	2.373^{b}±0.254	0.0020
Malonic acid (r)	µg g^{-1} FW	0.137±0.069	bDL	bDL	0.049±0.069	0.0967
Acetic acid (r)	µg g^{-1} FW	1.262±0.389	0.377±0.049	0.057±0.081	1.865±1.037	0.0771
Citric acid (r)	µg g^{-1} FW	bDL	0.368±0.328	0.187±0.021	bDL	0.2176
Fumaric acid (r)	µg g^{-1} FW	0.033^{a}±0.004	bDL	0.017^{b}±0.002	bDL	0.0092
Succinic acid (r)	µg g^{-1} FW	0.556^{b}±0.155	1.535^{a}±0.382	0.162^{c}±0.084	bDL	0.0060
LMWOAs (sum) (r)	µg g^{-1} FW	3.915^{b}±1.117	4.223^{b}±0.313	8.460^{a}±1.388	4.667^{b}±1.651	0.0450

Data presented as mean value ±SD (n=3); TPC – total phenolic compounds, RSC – relative scavenging capacity, 2,5-DHBA – 2,5-dihydroxybenzoic acid, 4-HBA – 4-hydroxybenzoic acid, C6-C1 – hydroxybenzoic acids, C6-C3 – phenylopropanoids, C6-C3-C6 – flavonoids, LMWOAs – low-molecular-weight organic acids (sum of detected compounds), l – leaves, r – roots, FW – fresh weight, bDL – below detection limit; identical superscripts denote no significant differences between means according to a post-hoc Fisher's Least Significant Difference (LSD) test following one-way ANOVA at α=95%; *p* – empirical level of significance
